# EHBP1L1 Drives Immune Evasion in Renal Cell Carcinoma through Binding and Stabilizing JAK1

**DOI:** 10.1002/advs.202206792

**Published:** 2023-02-12

**Authors:** Yihui Pan, Guannan Shu, Liangmin Fu, Kangbo Huang, Xinwei Zhou, Chengpeng Gui, Huashan Liu, Xiaohan Jin, Minyu Chen, Pengju Li, Junjie Cen, Zihao Feng, Jun Lu, Zhenhua Chen, Jiaying Li, Quanhui Xu, Yinghan Wang, Hui Liang, Zhu Wang, Qiong Deng, Wei Chen, Junhang Luo, Jiefeng Yang, Jiaxing Zhang, Jinhuan Wei

**Affiliations:** ^1^ Department of Urology The First Affiliated Hospital Sun Yat‐sen University Guangzhou 510080 China; ^2^ Department of Urology The Third Affiliated Hospital Soochow University Changzhou Jiangsu 213003 China; ^3^ Sun Yat‐sen University Cancer Center State Key Laboratory of Oncology in South China Collaborative Innovation Center for Cancer Medicine Guangzhou 510060 China; ^4^ Department of Urology Sun Yat‐sen University Cancer Center Guangzhou 510060 China; ^5^ Department of Colorectal Surgery and Guangdong Provincial Key Laboratory of Colorectal and Pelvic Floor Diseases The Sixth Affiliated Hospital Sun Yat‐sen University Guangzhou 510655 China; ^6^ Department of Urology Affiliated Longhua People's Hospital Southern Medical University Shenzhen 518109 China; ^7^ Department of Oncology The First Affiliated Hospital Sun Yat‐sen University Guangzhou 510080 China

**Keywords:** EHBP1L1, IFN‐*γ*/JAK1/STAT1/PD‐L1 signaling, immune evasion, renal cell carcinoma

## Abstract

High lymphocyte infiltration and immunosuppression characterize the tumor microenvironment (TME) in renal cell carcinoma (RCC). There is an urgent need to elucidate how tumor cells escape the immune attack and to develop novel therapeutic targets to enhance the efficacy of immune checkpoint blockade (ICB) in RCC. Overactivated IFN‐*γ*‐induced JAK/STAT signaling involves in such TME, but the underlying mechanisms remain elusive. Here, EH domain‐binding protein 1‐like protein 1 (EHBP1L1) is identified as a crucial mediator of IFN‐*γ*/JAK1/STAT1/PD‐L1 signaling in RCC. EHBP1L1 is highly expressed in RCC, and high EHBP1L1 expression levels are correlated with poor prognosis and resistance to ICB. EHBP1L1 depletion significantly inhibits tumor growth, which is attributed to enhanced CD8^+^ T cell‐mediated antitumor immunity. Mechanistically, EHBP1L1 interacts with and stabilizes JAK1. By competing with SOCS1, EHBP1L1 protects JAK1 from proteasomal degradation, which leads to elevated JAK1 protein levels and JAK1/STAT1/PD‐L1 signaling activity, thereby forming an immunosuppressive TME. Furthermore, the combination of EHBP1L1 inhibition and ICB reprograms the immunosuppressive TME and prevents tumor immune evasion, thus significantly reinforcing the therapeutic efficacy of ICB in RCC patient‐derived xenograft (PDX) models. These findings reveal the vital role of EHBP1L1 in immune evasion in RCC, which may be a potential complement for ICB therapy.

## Graphical abstract

Herein the authors identify EH domain‐binding protein 1‐like protein 1(EHBP1L1) as a key regulator of immune escape in renal cell carcinoma (RCC). EHBP1L1 interacts with and stabilizes Janus kinase 1 (JAK1), thus significantly inhibiting tumor growth and enhancing anti‐tumor immune response. This work reveals a potential therapeutic target for improving the efficacy of cancer immunotherapy in RCC patients.

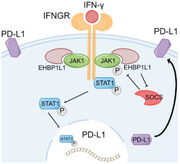

## 1 Introduction

Globally, an estimated 338 000 patients are diagnosed with renal cell carcinoma (RCC) every year, and 144 000 RCC patients die every year.^[^ ^1^ ^]^ It is expected that RCC will comprise approximately 4.1% of all new cancer cases in the USA in 2022, showing an upward trend year by year.^[^ ^2^ ^]^ Over 70% of all RCCs have a histology of clear cell RCC (ccRCC), followed by papillary and chromophobe RCCs.^[^ ^3^, ^4^ ^]^ The treatment strategies for advanced RCC have changed dramatically over the past few years. The combination of immunotherapy and targeted therapy has become the preferred regimen in the first‐line therapy of advanced RCC.^[^ ^5^, ^6^, ^7^, ^8^ ^]^ However, as a result of intrinsic or acquired resistance to immune checkpoint blockade (ICB), most RCC patients do not benefit from a long‐lasting response. Thus, identification of the potential drivers of immune evasion in RCC is urgently needed, and there is an unmet demand for therapeutic targets to sensitize RCC patients to ICB therapy.

RCC has long been considered an immunogenic cancer that is frequently infiltrated by immune cells.^[^ ^9^, ^10^ ^]^ A comprehensive study using mass cytometry to perform in‐depth immune profiling of the primary ccRCC tumor microenvironment (TME) has shown that CD8^+^ PD‐1^+^ T cells extensively infiltrate ccRCC and that the expression of T cell exhaustion markers is related to survival outcomes.^[^ ^11^ ^]^ Furthermore, a proteogenomic analysis of ccRCC from 232 Chinese patients found that the GP1 subtype, which has the strongest immunosuppression characteristics, is associated with the worst clinical outcome.^[^ ^12^ ^]^ These findings indicate that immune escape is common in RCC patients and is vital for long‐term survival.

Programmed cell death‐1 (PD‐1) and programmed death‐ligand 1 (PD‐L1) are classical immune checkpoints during tumor immune escape. Inhibition of the PD‐1/PD‐L1 axis restores T‐cell exhaustion and eradicates tumor cells.^[^ ^13^ ^]^ The interferon (IFN)‐*γ*/Janus kinase (JAK)/signal transducer and activator of transcription (STAT) signaling pathway, which is induced by extracellular IFN‐*γ*, has been widely recognized as the predominant inducer of PD‐L1.^[^ ^13^, ^14^, ^15^ ^]^ After IFN‐*γ* binds to its receptor, trans‐activated JAKs (JAK1 and JAK2) promote tyrosine phosphorylation of STATs (mainly STAT1). Phosphorylated STAT1 translocates to the nucleus, along with its downstream component interferon‐responsive factor‐1 (IRF‐1), binds to the *PD‐L1* promoter and upregulates the expression of PD‐L1.^[^ ^16^, ^17^ ^]^ Tumors with JAK1/2 mutations may develop intrinsic or acquired resistance, suggesting that the IFN‐*γ*/JAK/STAT pathway is critical for the efficacy of ICB.^[^ ^18^, ^19^ ^]^ Several studies have reported that this pathway is closely linked to immunotherapy responsiveness.^[^ ^20^, ^21^ ^]^ For instance, PBRM1 mutations impair the binding of BRG1 to the IFN‐*γ* receptor 2 promoter, subsequently inhibiting the IFN‐*γ*/STAT1 signaling and inducing ICB resistance.^[^ ^22^ ^]^ However, the underlying mechanism of overactivated IFN‐*γ*/STAT1 signaling in RCC remains unclear.

EH domain‐binding protein 1‐like protein 1 (EHBP1L1) is a paralog of EHBP1, and few studies on EHBP1L1 have been reported to date. EHBP1L1 may play a role in vesicular trafficking via interacting with the Rab family, including Rab8 and Rab10.^[^ ^23^, ^24^ ^]^ Our previous study reported that EHBP1L1 is correlated with poor prognosis in RCC.^[^ ^25^ ^]^ In the present study, we identify EHBP1L1 as a key regulator of IFN‐*γ*/JAK1/STAT1/PD‐L1 signaling, and we demonstrate that EHBP1L1 interacts with JAK1 and increases JAK1 stability. The EHBP1L1‐JAK1 interaction protects JAK1 from ubiquitination and proteasomal degradation. Moreover, the combined inhibition of EHBP1L1 and immune checkpoint exhibits an enhanced antitumor effect in RCC preclinical patient‐derived xenograft (PDX) models compared to monotherapy. Overall, the present study reveals the critical effect of EHBP1L1 in promoting immune evasion through upregulating JAK1/STAT1/IFN‐*γ* signaling, which may be a potential complement for ICB treatment in RCC.

## 2 Results

### 2.1 EHBP1L1 Is a Prognostic Factor and Is Associated with Immunosuppression in RCC

**Figure 1:**
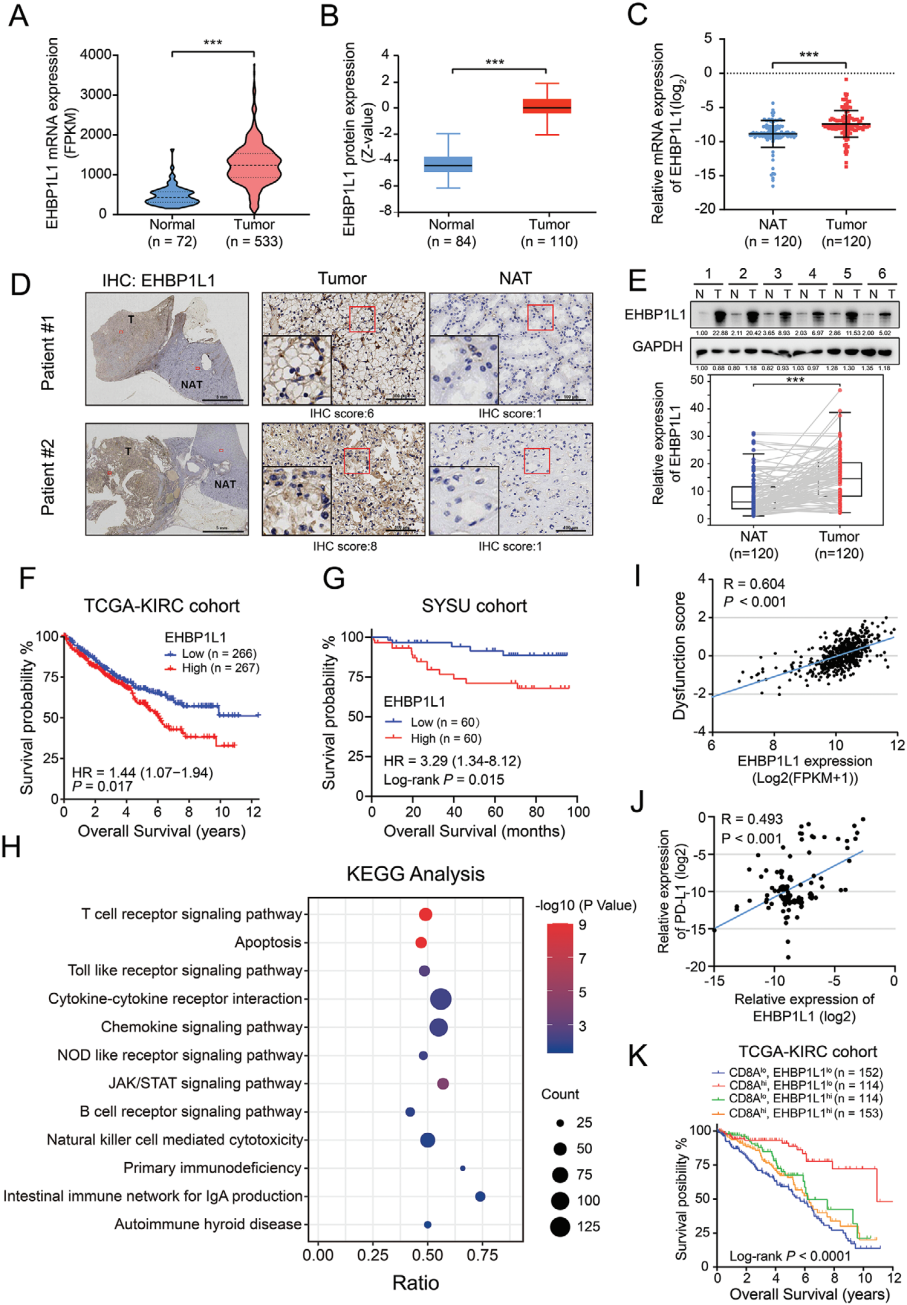
EHBP1L1 is upregulated in RCC and is correlated with immunosuppression in RCC. A) EHBP1L1 mRNA levels in ccRCC tumor tissues and normal tissues from TCGA database. B) Mass spectrometry‐based proteomic profiling of EHBP1L1 expression in various tumor types and peritumor normal tissues as identified through CPTAC database. C) Relative RNA expression of EHBP1L1 in 120 pairs of human RCC tumors (T) and matched normal adjacent tissues (NAT) according to qRT‐PCR. D) Representative immunohistochemical (IHC) images showing the expression of EHBP1L1 in tumors (T) and matched normal adjacent tissues (NAT). E) Representative western blot (top panel) and statistical analysis (bottom panel) of EHBP1L1 protein expression levels in 120 pairs of human RCC tumors (T) and normal adjacent tissues (NAT). F) OS of RCC patients with low (*n* = 266) or high (*n* = 267) EHBP1L1 expression in the TCGA‐KIRC cohort. G) OS of RCC patients with low (*n* = 60) or high (*n* = 60) EHBP1L1 expression in our independent cohort. H) KEGG analysis indicated that high expression of EHBP1L1 was associated with pathways related to antitumor immune function in TCGA‐KIRC dataset. The top 50% expression of EHBP1L1 was defined as the high expression group. The bottom 50% expression of EHBP1L1 was defined as the low expression group. I) TIDE analysis revealed that EHBP1L1 was positively associated with immune dysfunction score in TCGA‐KIRC dataset. J) qPCR analysis showed that EHBP1L1 expression was positively correlated with PD‐L1 expression in the SYSU RCC cohort *(n* = 120). K) Overall survival of RCC patients in the TCGA‐KIRC cohort, stratified by both EHBP1L1 and CD8A expression. All experiments were performed with three independent biological replicates.

Our previous study identified an EHBP1L1‐based classifier for ccRCC prognosis.^[^ ^25^ ^]^ To further investigate the potential role of EHBP1L1 in tumors, we assessed EHBP1L1 expression levels in tumor and normal tissues using the Cancer Genome Atlas (TCGA) database. Bioinformatic analyses suggested that EHBP1L1 was significantly up‐regulated in kidney renal clear cell carcinoma (KIRC) (**Figure** **1**A). Further analysis of the Clinical Proteomic Tumor Analysis Consortium (CPTAC) database indicated that the EHBP1L1 protein level was significantly elevated in ccRCC (Figure 1B). The elevated expression of EHBP1L1 was further validated in paired RCC tissue samples by quantitative real‐time polymerase chain reaction (qRT‐PCR), immunohistochemistry staining, and western blot analyses (Figure 1C–E). Kaplan–Meier survival curves showed that high expression of EHBP1L1 in RCC patients was correlated with poor overall survival (OS) in the TCGA‐KIRC cohort (Figure 1F). These findings were further confirmed in an independent RCC cohort from Sun Yat‐sen University (SYSU), in which EHBP1L1 expression was detected by immunohistochemistry (IHC) staining (Figure 1G; Tables S1 and S2 and Figure S1A, Supporting Information).

To examine the detailed role of EHBP1L1 in RCC, we analyzed the RNA expression profiles from TCGA‐KIRC database. Interestingly, KEGG analysis based on EHBP1L1 expressions indicated that EHBP1L1 mainly participated in pathways related to antitumor immune response (Figure 1H). Although EHBP1L1 expression did not globally alter the composition of immune cells in the TME (Figure S1B,C, Supporting Information), further analysis revealed that the expression of EHBP1L1 was positively associated with immune dysfunction score as calculated by tumor immune dysfunction and exclusion (TIDE) (Figure 1I). Consistently, EHBP1L1 was positively correlated with PD‐L1 expression in the SYSU RCC cohort (Figure 1J). Additionally, given the critical role of CD8^+^ T cells in antitumor immune response, we next investigated the prognostic relevance of EHBP1L1 in patients with high or low CD8^+^ T cells infiltration (indicated by CD8A expression) in the TCGA KIRC cohort. To our surprise, though patients did not benefit from CD8^+^ T cells infiltration, patients with low EHBP1L1 expression and high CD8^+^ T cells infiltration exhibited the best survival (Figure 1K; Figure S1D, Supporting Information). Furthermore, in a metastatic ccRCC patient cohort who received ICB treatment,^[^ ^26^ ^]^ we found that the high cytotoxic T lymphocyte (CTL) score was correlated with a better OS but that high expression of EHBP1L1 abolished this survival benefit (Figure S1E, Supporting Information). Taken together, these results indicate that elevated expression of EHBP1L1 is unfavorable for patients with RCC and is associated with a suppressed immune response.

### 2.2 EHBP1L1 Depletion Enhances the Antitumor Immune Response in RCC

**Figure 2:**
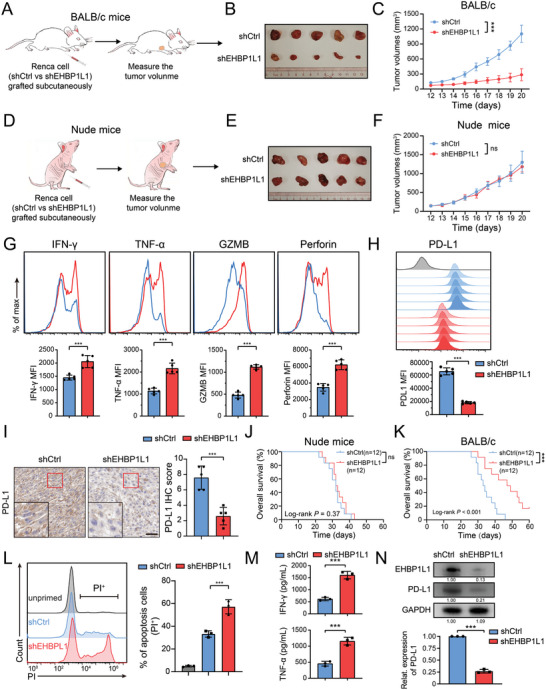
EHBP1L1 deficiency improves antitumor immunity in murine and human RCC. A,D) Schematic protocols of Renca cells with or without EHBP1L1 knockdown subcutaneously injected into A) immunocompetent BALB/c and D) immunodeficient nude mice. B,C) Representative images of B) tumors and C) growth curves of indicated Renca tumors in BALB/c mice (*n* = 5 per group). E,F) Representative images of tumors (B) and growth curves (C) of indicated Renca tumors in nude mice (*n* = 5 per group). G) Flow cytometry analysis of IFN‐*γ*, TNF‐*α*, GZMB and Perforin in CD8^+^ T cells isolated from indicated Renca tumors in BALB/c mice. H) Representative flow cytometry histograms (top panel) and statistical analysis (bottom panel) of cell surface PD‐L1 on indicated Renca cells in tumors. I) Representative IHC staining images of PD‐L1 in indicated Renca tumors in BALB/c mice (left panel) and statistical analysis of IHC scores (right panel). J,K) Survival curves of K) immunocompetent BALB/c and J) immunodeficient nude mice orthotopically implanted with indicated Renca cells (*n* = 12 per group). L) Representative flow cytometry histograms (left panel) and statistical analysis (right panel) of apoptosis rate (PI^+^) of primary kidney tumor cells with or without EHBP1L1 knockdown cocultured with primary kidney tumor‐specific CD8^+^ T cells. M) ELISA analysis of the IFN‐γ and TNF‐*α* levels in supernatant from primary kidney tumor cells with or without EHBP1L1 knockdown cocultured with primary kidney tumor‐specific CD8^+^ T cells. N) Representative western blot (top panel) and statistical analysis (bottom panel) of EHBP1L1 and PD‐L1 protein expression in primary kidney tumor cells with EHBP1L1 knockdown. A–F) Data represent one independent experiment with 5 mice per group. G–I) Each experiment was repeated three times with 5 mice per group, and data shown are representative of three independent experiments. J,K) Data represent one independent experiment with 12 mice per group. L–N) Data represent three independent biological replicates.

We further sought to confirm the immunosuppressive role of EHBP1L1 as indicated by clinical data. Single‐cell RNA sequencing (scRNA‐Seq) analysis of ccRCC tumor tissues revealed that EHBP1L1 was expressed in tumor cells rather than other cell types (Figure S2A, Supporting Information).^[^ ^27^ ^]^ We then specifically knocked down EHBP1L1 in the Renca murine RCC cell line by lentiviral short‐hairpin RNAs (shRNA) (Figure S2B, Supporting Information). EHBP1L1 deficiency did not significantly affect the proliferation of Renca cells in vitro (Figure S2C, Supporting Information). Compared to control cells (shCtrl), however, EHBP1L1 deficiency (shEHBP1L1) significantly inhibited Renca‐derived tumor growth in immunocompetent BALB/c mice (**Figure** **2**A–C) but not in immunodeficient nude mice (Figure 2D–F). These results indicate that EHBP1L1 does not significantly affect RCC proliferation and that it regulates tumor growth mainly by mediating the interaction of tumor cells with immune cells.

To determine whether EHBP1L1 deletion augments the cytotoxicity of T cells in vivo, we isolated CD8^+^ T cells from the aforementioned Renca tumors in BALB/c mice, and the results indicated that the effector function of CD8^+^ T cells was enhanced in the shEHBP1L1 group (Figure 2G). Moreover, flow cytometry analysis and IHC staining demonstrated a reduction of surface and total PD‐L1 protein levels in shEHBP1L1 Renca cell‐derived tumors (Figure 2H,I). Similar results were also observed in an orthotopic tumor model, in which remarkably improved survival was observed in immunocompetent mice implanted with shEHBP1L1 Renca cells but not in immunodeficient nude mice implanted with shEHBP1L1 Renca cells (Figure 2J,K; Figure S2D,E, Supporting Information). Collectively, the Renca tumor model uncovers a critical role of EHBP1L1 in the regulation of antitumor immunity in murine RCC.

To investigate the role of EHBP1L1 in more clinically relevant models, we isolated primary tumor cells from HLA‐A2^+^ RCC patients (Figure S2F, Supporting Information). Tumor‐specific CD8^+^ T cells were generated by activating T cells with dendritic cells (DCs) pulsed with primary tumor lysates as previously described (Figure S2G, Supporting Information).^[^ ^28^, ^29^ ^]^ EHBP1L1 silencing in primary tumor cells significantly enhanced the cytotoxicity of primary RCC tumor antigen‐activated CD8^+^ T cells to target tumor cells (Figure 2L). Consistently, EHBP1L1 loss increased production of cytokines (Figure 2M). Moreover, PD‐L1 was downregulated in primary RCC cells with EHBP1L1 knockdown (Figure 2N). Altogether, these findings demonstrate that high expression of EHBP1L1 in human RCC cells inhibits antitumor immune response.

### 2.3 Expression of EHBP1L1 in RCC Inhibits Intratumoral CD8^+^ T Cells Function and Drives Them toward a Dysfunctional State

**Figure 3:**
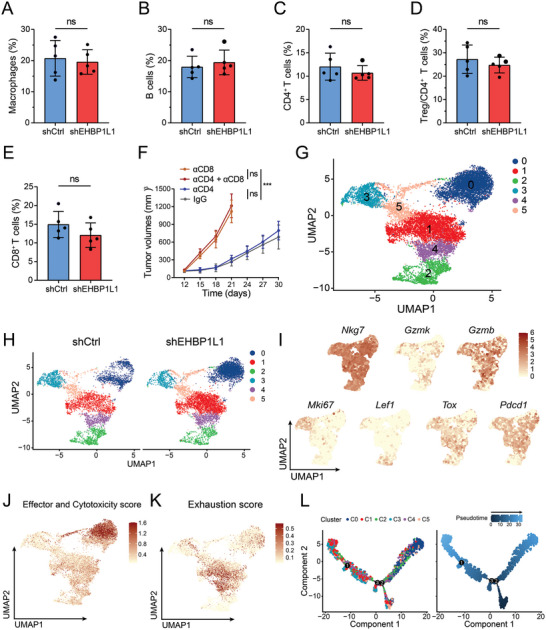
Effect of RCC EHBP1L1 expression in CD8^+^ T cells at single‐cell resolution in the tumor microenvironment. A–E) Percentage of A) CD11b^+^F4/80^+^ macrophages, B) CD3^−^CD19^+^ B cells, C) CD3^+^CD4^+^ T cells, D) CD3^+^CD4^+^Foxp3^+^ Treg and E) CD3^+^CD8^+^ T cells in CD45^+^ immune cells detected by flow cytometry infiltrating in shCtrl and shEHBP1L1 Renca tumors in BALB/c mice. F) Tumor growth curves of shEHBP1L1 Renca tumors treated with *α*CD8 or *α*CD4 antibody in BALB/c mice (*n* = 5 per group). G) Uniform manifold approximation and projection (UMAP) plots of tumor‐infiltrating CD8^+^ T cells in shCtrl and shEHBP1L1 Renca tumors in BALB/c mice. H) Distribution of tumor‐infiltrating CD8^+^ T cell clusters across shCtrl and shEHBP1L1 Renca tumors in BALB/c mice. I) The *Nkg7*, *Gzmk*, *Gzmb*, *Mki67*, *Lef1*, *Tox*, and *Pdcd1* expression levels in CD8^+^ T cell clusters are indicated in the UMAP plot. J,K) Enrichment pattern of Effector and Cytotoxicity score and Exhaustion score in the UMAP plot. L) The potential developmental trajectory of CD8^+^ T cell clusters in tumor microenvironment. Each dot represents a single cell, colored according to clusters (left) or pseudotime (right). A–E) Each experiment was repeated three times with 5 mice per group, and data shown are representative of three independent experiments. F) Data represent one independent experiment with 5 mice per group.

To determine which immune cell types contribute to enhanced antitumor immunity upon EHBP1L1 depletion, we analyzed subpopulations of CD45^+^ immune cells in shCtrl and shEHBP1L1 Renca tumors (Figure S3A, Supporting Information). However, no significant differences in the percentages of macrophages, B cells, CD4^+^ T cells, regulatory T cells (Tregs) and CD8^+^ T cells were observed between the shCtrl and shEHBP1L1 group (**Figure** **3**A–E). Given that the PD‐1/PD‐L1 axis accelerates tumor growth mainly by repressing the antitumor killing activity of T cells in the TME and that PD‐L1 was down‐regulated upon EHBP1L1 loss, we depleted CD4^+^ T cells, CD8^+^ T cells, or both to further validate whether T cell populations contribute to the improved antitumor immunity upon EHBP1L1 loss. CD4^+^ and CD8^+^ T cells in the Renca tumor mouse model were depleted with anti‐CD4‐ or anti‐CD8‐neutralizing antibodies. We found that depletion of CD8^+^ T cells or both CD4^+^ and CD8^+^ T cells significantly reversed the tumor regression induced by EHBP1L1 knockdown, whereas depletion of CD4^+^ T cells alone, had no such effect similar to the IgG control (Figure 3F; Figure S3B, Supporting Information). Furthermore, we found that in the TCGA‐KIRC cohort, low EHBP1L1 expression levels were associated with increased cytotoxicity of CD8^+^ T cells (Figure S3C, Supporting Information). These results indicate that EHBP1L1 depletion in RCC cells may enhance antitumor immunity in a CD8^+^ T cell‐dependent manner.

To better understand the effect of EHBP1L1 expression in tumor cells on CD8^+^ tumor‐infiltrating lymphocytes (TILs), we performed scRNA‐seq on CD8^+^ TILs that were FACS‐sorted from shCtrl and shEHBP1L1 Renca tumors. Expression of canonical T cell markers validated the successful sorting of CD8^+^ TILs (Figure S3D, Supporting Information). Cell clustering analysis revealed 6 subpopulations of CD8^+^ T cells as illustrated in a Uniform Manifold Approximation and Projection (UMAP) plot (Figure 3G). Cluster 2 specifically expressed naïve markers like *Lef1* and *Ccr7* and was composed of naïve T cells. Cluster 1 was characterized by high expression of genes related to T cell exhaustion and represented exhausted CD8^+^ T cells (T_ex_). Cluster 0 expressed high levels of cytotoxic effectors and low levels of exhausted genes and was referred to as effector T cells (T_eff_) (Figure S3E, Supporting Information). We next compared the abundance of each subpopulation in shCtrl and shEHBP1L1 Renca tumors. We noticed that CD8^+^ T_eff_ subpopulation (Cluster 0) increased in EHBP1L1 lose tumors, whereas CD8^+^ T_ex_ subpopulation (Cluster 1) diminished (Figure 3H; Figure S3F, Supporting Information). Expression levels of cytotoxicity and exhaustion genes and visualization of the cytotoxic and exhaustion scores further validated elevated effector function and diminished dysfunction of CD8^+^ T cells upon EHBP1L1 depletion in tumors (Figure 3I–K). To further reveal the intrinsic ontogeny of CD8^+^ T cells, we applied the Monocle 2 algorithm to construct their potential developmental trajectories.^[^ ^30^ ^]^ Interestingly, two major evolution branches were observed, and cluster 0 and 1 were positioned at the end of different branches (Figure 3L). This analysis demonstrated distinct differentiation trajectories of CD8^+^ TIL in tumor microenvironment by EHBP1L1, in which CD8^+^ T cells infiltrating in EHBP1L1 loss tumors were more likely to differentiate into T_eff_ rather than T_ex_. Together, these results suggest that EHBP1L1 expression in tumors suppresses the effecter function of CD8^+^ T cells and directs T cell differentiation toward a dysfunctional trajectory.

### 2.4 EHBP1L1 Regulates PD‐L1 Expression through the JAK1/STAT1 Signaling Pathway in RCC

**Figure 4:**
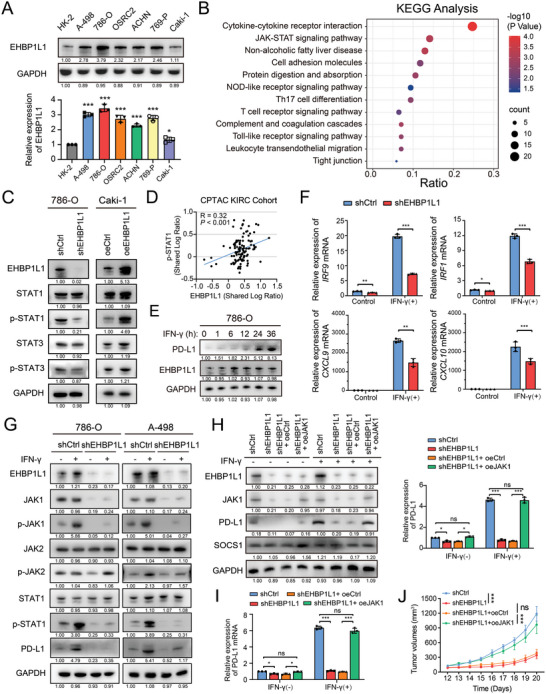
Loss of EHBP1L1 impairs JAK1‐STAT signaling activity in RCC cells. A) Representative western blot (top panel) and statistical analysis (bottom panel) of EHBP1L1 protein expression levels in the immortalized HK‐2 renal epithelial cell line and RCC cell lines (A‐498, 786‐O, OSRC2, ACHN, 769‐P, and Caki‐1). GAPDH was used as a loading control. B) KEGG analysis showed the significantly altered signaling pathways after EHBP1L1 silencing in 786‐O cells. C) Representative western blot of STAT1, STAT3, p‐STAT1, and p‐STAT3 protein expression levels in 786‐O cells with EHBP1L1 knockdown (left panel) and in Caki‐1 cells with EHBP1L1 overexpression (right panel). D) Correlation analysis between EHBP1L1 and p‐STAT1 protein levels in the CPTAC KIRC cohort. E) Representative western blot showing PD‐L1 protein expression levels after IFN‐*γ* stimulation in 786‐O cells. Cells were treated with 100 U mL^‐1^ IFN‐*γ* for the indicated times. F) mRNA expression of JAK1/STAT1 target genes in shCtrl and shEHBP1L1 786‐O cells with or without IFN‐*γ* stimulation. Cells were treated with 100 U mL^‐1^ IFN‐*γ* for the indicated times. G) Representative western blot showing JAK1, p‐JAK1, JAK2, p‐JAK2, STAT1, p‐STAT1 and PD‐L1 protein expression levels in 786‐O (left panel) and A‐498 (right panel) cells with EHBP1L1 knockdown. Cells were stimulated with or without 100 U mL^‐1^ IFN‐*γ* for 8 h. H) Effects of JAK1 overexpression on PD‐L1 protein expression in shCtrl and shEHBP1L1 786‐O cells with or without IFN‐*γ* stimulation. I) Effects of JAK1 overexpression on PD‐L1 mRNA expression in shCtrl and shEHBP1L1 786‐O cells with or without IFN‐*γ* stimulation. J) Tumor growth curves of indicated Renca tumors in BALB/c mice (*n* = 5 per group). A–I) All experiments were performed with three independent biological replicates, and data shown are representative of three independent experiments. J) Data represent one independent experiment with 5 mice per group .

We next investigated the biological mechanism of EHBP1L1 in regulating PD‐L1 expression. To select the most suitable RCC cell lines, we verified EHBP1L1 expression in a panel of RCC cell lines (A‐498, 786‐O, OSRC2, ACHN, 769‐P and Caki‐1) and an immortalized renal epithelial cell line (HK‐2). Western blot and qRT‐PCR analyses showed that EHBP1L1 protein and mRNA levels, respectively, were markedly upregulated in all RCC cell lines compared to primary normal HK2 cells (**Figure** **4**A; Figure S4A, Supporting Information). Based on the endogenous EHBP1L1 levels, we knocked down EHBP1L1 in 786‐O and A‐498 cells, and we overexpressed EHBP1L1 in Caki‐1 cells. RNA‐sequencing (RNA‐seq) of 786‐O cells indicated significant transcriptional changes when EHBP1L1 was knocked down (Figure S4B, Supporting Information). KEGG analysis revealed that differentially expressed genes were functionally enriched in pathways related to immune function (Figure 4B). Among them, the JAK/STAT pathway has been shown in numerous studies to be involved in immunotherapy responsiveness.^[^ ^18^, ^19^, ^20^, ^21^ ^]^ The RNA‐seq results were further validated by western blot analysis. Knockdown of EHBP1L1 significantly decreased the protein levels of p‐STAT1, while EHBP1L1 overexpression significantly elevated p‐STAT1 protein levels (Figure 4C). The above findings were further confirmed in the CPTAC KIRC cohort (Figure 4D). Thus, these results suggest that EHBP1L1 enhances the JAK/STAT1 signaling pathway in RCC cells.

IFN‐*γ* stimulation induces activation of the JAK/STAT1 signaling pathway and leads to upregulation of PD‐L1, which is critical for de novo resistance or acquired resistance to ICB therapy. Upon IFN‐*γ* stimulation, both human and murine RCC cells exhibited a significant increase in PD‐L1 and STAT1 target genes expression (Figure 4E,F; Figure S4C, Supporting Information). Moreover, EHBP1L1 knockdown remarkably decreased the total protein level of JAK1 and inhibited IFN‐*γ*‐induced JAK1/STAT1 target genes expression, JAK1 and STAT1 phosphorylation as well as the upregulation of PD‐L1 (Figure 4F,G; Figure S4D, Supporting Information). The total and phosphorylation levels of JAK2, another upstream kinase of STAT1, were unaffected by EHBP1L1 knockdown. To determine whether downregulation of PD‐L1 is attributed to decreased JAK1 levels in EHBP1L1 knockdown cells, a rescue experiment with JAK1 overexpression was performed. JAK1 overexpression restored PD‐L1 expression in EHBP1L1 knockdown cells (Figure 4H,I; Figure S4E,F, Supporting Information), and promoted tumor growth in BALB/c mice (Figure 4J). Taken together, these results demonstrate that EHBP1L1 enhances the activity of JAK1‐STAT1 signaling to promote the expression of PD‐L1 and thus promotes tumor growth.

### 2.5 Identification of JAK1 as an Interacting Protein of EHBP1L1

**Figure 5:**
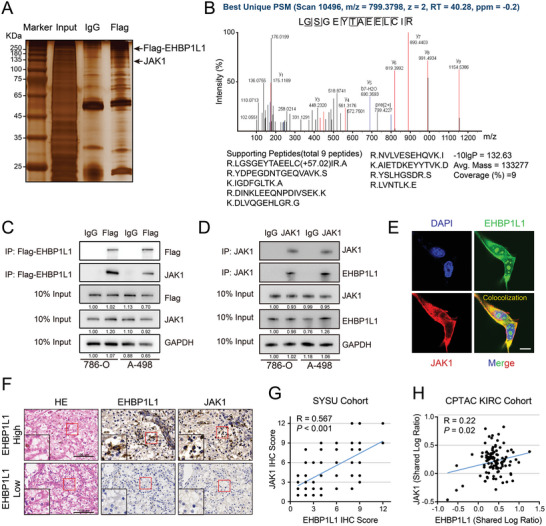
EHBP1L1 interacts with JAK1. A) Representative silver staining gel showing the protein bands (indicated with arrowheads) specifically presented in the Flag‐EHBP1L1 pulldown sample that were subjected to LC‐MS/MS analysis. B) Mass spectrometry identification of JAK1, which was pulled down from 786‐O lysates by a Flag antibody for Flag‐EHBP1L1. C) Representative western blot showing the co‐IP of overexpressed EHBP1L1‐Flag and endogenous JAK1 in both 786‐O and A‐498 cells. D) Representative western blot showing the co‐IP of endogenous JAK1 and EHBP1L1 in both 786‐O and A‐498 cells. E) Representative immunofluorescent images of endogenous JAK1 and EHBP1L1 in 786‐O cells. Scale bar = 10 µm. F) Serial sections of tumor tissues in 120 RCC patients stained by HE, EHBP1L1 and JAK1. Scale bar = 100 µm. G) Correlation analysis between EHBP1L1 and JAK1 protein levels (IHC score) in 120 human RCC samples. H) Correlation analysis between EHBP1L1 and JAK1 protein levels in the CPTAC KIRC cohort. All experiments were performed with three independent biological replicates, and data shown are representative of three independent experiments.

To elucidate the molecular mechanisms of EHBP1L1 in regulating the JAK1/STAT1 signaling pathway, Flag‐tagged EHBP1L1 was stably overexpressed in 786‐O cells and pulled down, and the global protein interactome of EHBP1L1 was detected using liquid chromatography coupled with mass spectrometry (LC‐MS/MS) (**Figure** **5**A). To determine the proteins that specifically bind to EHBP1L1, we compared the Flag‐EHBP1L1‐binding proteins with IgG‐binding proteins. The proteins that bound to IgG were excluded, and the remaining proteins were ranked by MS score. Among them, JAK1 was one of the top five candidates to specifically associate with EHBP1L1 (Figure 5B; Figure S5A, Supporting Information). To confirm the interaction between EHBP1L1 and JAK1, a coimmunoprecipitation (co‐IP) assay was performed. Overexpressed Flag‐tagged EHBP1L1 co‐immunoprecipitated with endogenous JAK1 in both 786‐O and A‐498 cells (Figure 5C). Moreover, the interaction between endogenous EHBP1L1 and JAK1 was confirmed in 786‐O and A‐498 cells using specific monoclonal antibodies (Figure 5D).

Immunofluorescence colocalization analysis showed that EHBP1L1 and JAK1 colocalized mainly in the cytoplasm in 786‐O cells, indicating that the EHBP1L1‐JAK1 complex may function in the cytoplasm (Figure 5E). We then examined the protein expression level of these two proteins by IHC in RCC samples, which indicated that EHBP1L1 expression was correlated with JAK1 expression (Figure 5F,G). These results were further validated by the publicly available mass spectrometry‐based proteomics database, CPTAC, in which EHBP1L1 expression was positively correlated with JAK1 expression in the KIRC cohort (Figure 5H). Furthermore, in a panel of representative tumor cells (HCT‐116, A549, MAD‐MB231, AGS, SK‐Mel‐28, HeLa and Huh7), EHBP1L1 protein expression level was positively related to that of JAK1 (Figure S5B,C, Supporting Information), suggesting that the EHBP1L1‐JAK1 axis is common among multiple tumor types. Thus, these results identify JAK1 as an interacting protein of EHBP1L1, providing substantial evidence to support a strong association between EHBP1L1 and JAK1.

### 2.6 EHBP1L1 Stabilizes JAK1 by Preventing SOCS1‐Mediated JAK1 Ubiquitination and Degradation

**Figure 6:**
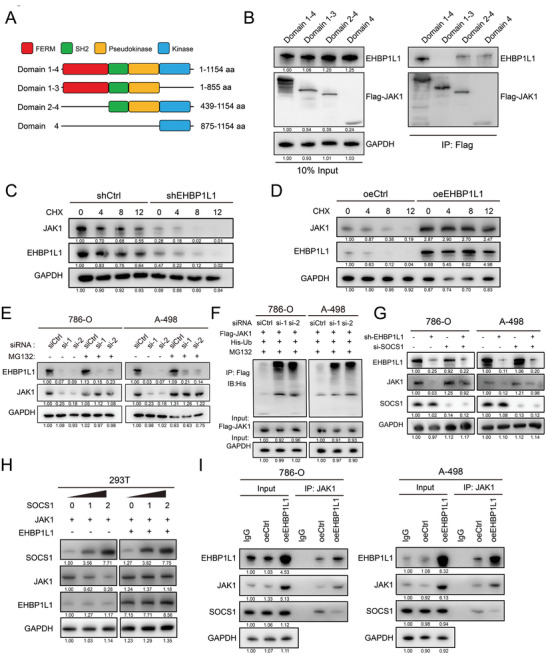
Binding of EHBP1L1 to JAK1 increases its stability by competing with SOCS1. A) Schematic representation of JAK1 and its truncated forms. Sequence and structure analyses indicate the presence of domain 1 (FERM domain), domain 2 (SH2 domain), domain 3 (Pseudokinase domain), and domain 4 (Kinase domain). aa, amino acid. B) Co‐IP showing the region of JAK1 bound to EHBP1L1. Total cell proteins from 786‐O cells transfected with the indicated constructs were subjected to immunoprecipitation with an anti‐Flag antibody (against JAK1) followed by immunoblotting with anti‐Flag or anti‐EHBP1L1 antibodies. C,D) Representative western blot from the CHX assay showing JAK1 protein stability after EHBP1L1 knockdown in C) 786‐O cells and D) EHBP1L1 overexpression in Caki‐1 cells. Cells were treated with 20 µg mL^‐1^ CHX for the indicated times. E) Representative western blot showing the reversal of JAK1 protein levels induced by the MG132 proteasome inhibitor in EHBP1L1‐deficient 786‐O cells (left panel) and A‐498 cells (right panel). F) Co‐IP assays showing that EHBP1L1 silencing promoted JAK1 ubiquitination levels. Total cell proteins from 786‐O cells (left panel) and A‐498 cells (right panel) transfected with Flag‐EHBP1L1 and His‐Ub followed by treatment with 20 × 10^‐6^ mp. r. MG132 for 12 h were subjected to IP assays with an anti‐Flag antibody. G) Representative western blot showing the rescue of JAK1 protein levels in the EHBP1L1 knockdown 786‐O and A‐498 cells when silencing SOCS1 (siSOCS1). H) Representative western blot showing the effect of exogenous EHBP1L1 and SOCS1 on the levels of exogenous JAK1 in the 293T cells transfected with indicated plasmids. I) Co‐IP assay showing the competition of EHBP1L1 with SOCS1 for binding to JAK1 in 786‐O cells (left panel) and A‐498 cells (right panel). All experiments were performed with three independent biological replicates, and data shown are representative of three independent experiments.

JAK1 is emerging as an important nonreceptor tyrosine kinase, and it is comprised of FERM (4.1 protein, ezrin, radixin, moesin), SH2‐like, pseudokinase, and kinase domains.^[^ ^16^ ^]^ To identify the regions of JAK1 responsible for interaction with EHBP1L1, we constructed a series of JAK1 deletion mutants harboring domains 1‐4 (1‐1154), domains 1‐3 (1‐855), domains 2‐4 (439‐1154) and domain 4 (875‐1154) (**Figure** **6**A), and we separately transfected each EHBP1L1 deletion mutant into 786‐O cells followed by co‐IP analysis. Interestingly, EHBP1L1 mainly interacted with the kinase domain of JAK1 (domain 4) but not the other domains (Figure 6B).

We then investigated the biological effect of the EHBP1L1‐JAK1 interaction on JAK1. Because the mRNA levels of JAK1 remained relatively unchanged upon EHBP1L1 knockdown (Figure S6A, Supporting Information), we hypothesized that the diminished JAK1 protein level observed in EHBP1L1 knockdown cells (Figure 4G) is due to the decreased stabilization of JAK1 protein. To confirm our hypothesis, we assessed JAK1 protein stability after blocking protein synthesis with cycloheximide (CHX). After CHX treatment, JAK1 was rapidly degraded in RCC cells with EHBP1L1 knockdown (Figure 6C; Figure S6B, Supporting Information), whereas JAK1 protein had a significantly increased half‐life in cells with EHBP1L1 overexpression (Figure 6D; Figure S6C, Supporting Information). Furthermore, the decreased protein expression of JAK1 in EHBP1L1 knockdown cells was recovered by the MG132 proteasome inhibitor (Figure 6E), indicating that EHBP1L1 stabilizes JAK1 in a proteasome‐dependent manner. In addition, an IP assay using an anti‐Flag antibody was conducted in cells co‐transfected with Flag‐tagged JAK1 and His‐Ub, and JAK1 ubiquitin levels were detected by western blot analysis. EHBP1L1 knockdown significantly elevated the poly‐ubiquitination of JAK1 in RCC cells (Figure 6F). These data suggest that EHBP1L1 promotes JAK1 stabilization by inhibiting its polyubiquitination degradation.

The suppressor of cytokine signaling (SOCS) family has been reported to promote the ubiquitination of JAK1 and inhibit JAK1 phosphorylation through binding to the GQM motif, which is located in the kinase domain (Figure S6D, Supporting Information). By recruiting Cullin5, the SOCS‐box domain of SOCS proteins functions as a ubiquitin ligase to ubiquitinate and degrade JAK1. Among the SOCS family members, SOCS1 is the most potent.^[^ ^31^, ^32^ ^]^ Here, we observed enhanced ubiquitination of JAK1 when silencing EHBP1L1, and EHBP1L1 bound directly to the kinase domain of JAK1. Therefore, we hypothesized that EHBP1L1 may prevent the ubiquitination of JAK1 via blocking its interaction with SOCS1. Silencing SOCS1 by a specific siRNA restored JAK1 levels in RCC cells with EHBP1L1 knockdown (Figure 6G). When SOCS1, JAK1 or EHBP1L1 were coexpressed in 293T cells, SOCS1 decreased JAK1 protein levels in a dose‐dependent manner, and the inhibitory effect of SOCS1 on JAK1 was antagonized by EHBP1L1 overexpression (Figure 6H). Furthermore, a co‐IP experiment was performed to evaluate the interaction among EHBP1L1, JAK1, and SOCS1, which demonstrated that EHBP1L1 competed with SOCS1 to bind to JAK1 (Figure 6I). Taken together, these findings reveal that EHBP1L1 competitively binds to the kinase domain of JAK1, thereby preventing the SOCS1‐mediated ubiquitination and degradation of JAK1.

### 2.7 Targeting EHBP1L1 Enhances the Efficacy of Tumor Immunotherapy in Human RCC PDX Models

**Figure 7:**
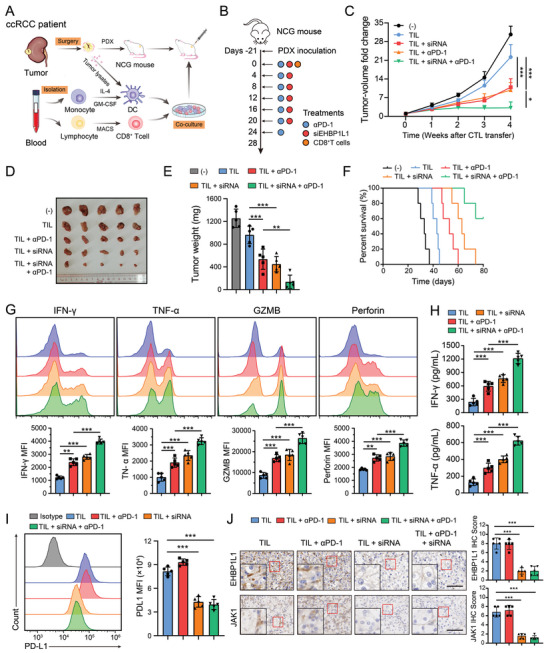
Therapeutic potential of EHBP1L1 knockdown and anti‐PD‐1 combination therapy in RCC PDX models. A) Schematic diagram of the generation and adoptive transfer of human RCC‐specific CD8^+^ T cells into NCG mice transplanted with RCC PDXs. B) Schematic diagram showing that RCC PDX mice were treated with adoptive T cell transfer, anti‐PD‐1 antibody and siRNA at the indicated time points. In the figure, ‐21 indicates the day of subcutaneous inoculation of RCC PDXs. C–F) The anti‐PD‐1 and siEHBP1L1 combination therapy synergistically suppressed the growth of tumors in RCC PDX mice (*n* = 5 per group). C) Tumor growth curves, D) tumor images, E) tumor weights, and F) survival curves. G) Flow cytometry analysis of IFN‐*γ*, TNF‐*α*, GZMB, and Perforin in CD8^+^ T cells isolated from PDX tumors with the indicated treatments. H) ELISA analysis of the IFN‐γ and TNF‐*α* levels in serum samples from PDX mice with the indicated treatments. I) Representative flow cytometry histograms (left panel) and statistical analysis (right panel) of cell surface PD‐L1 in tumors from PDX mice with the indicated treatments. J) Representative IHC staining images of JAK1 in tumors from PDX mice with the indicated treatments (left panel) and statistical analysis (right panel) of IHC scores. Scale bar = 100 µm. C–F) Data represent one independent experiment with 5 mice per group. G–J) Each experiment was repeated three times with 5 mice per group, and data shown are representative of three independent experiments.

Based on the above results, we sought to explore the possibility of targeting EHBP1L1 in human tumors to improve the therapeutic efficacy of cancer immunotherapy. To better mimic the TME of RCC, we utilized a preclinical model, in which immunocompromised NCG mice were implanted with RCC PDXs followed by tumor‐specific CD8^+^ T cell transfer (**Figure** **7**A).^[^ ^29^, ^33^ ^]^ A specific siRNA targeting EHBP1L1 was employed to investigate the effect of EHBP1L1 inhibition on anti‐PD‐1 therapy in a humanized immune system model (Figure 7B).

Treatment with siRNA effectively knocked down EHBP1L1 in PDX tumor tissues (Figure S7A, Supporting Information). Consistent with the above results, EHBP1L1 siRNA did not affect PDX tumor growth in NCG mice without immune reconstitution (Figure S7B, Supporting Information). Further analysis showed that although both EHBP1L1 siRNA and anti‐PD‐1 monotherapy inhibited tumor growth and prolonged OS, siEHBP1L1 and PD‐1 blockade combination therapy remarkably reduced tumor burden and improved OS compared to treatment with siEHBP1L1 or anti‐PD‐1 antibody alone (Figure 7C–F). Additionally, combination therapy significantly improved the effector function of CD8^+^ T cells as revealed by flow cytometry and enzyme‐linked immunosorbent assay (ELISA) analysis of cytolytic cytokines and granules (Figure 7G,H; Figure S7C, Supporting Information). In both siRNA groups, siEHBP1L1 treatment significantly reduced cell surface PD‐L1 expression in PDX tumors as revealed by flow cytometry analysis (Figure 7I). As expected, IHC staining of PDX tumor tissues indicated that siEHBP1L1 treatment led to decreased JAK1 protein level (Figure 7J). Taken together, these findings indicate that EHBP1L1 inhibition enhances the therapeutic effects of PD‐1 blockade and may be a potential target for improving cancer immunotherapy efficacy in RCC.

## 3 Conclusion

TILs have been reported to be associated with survival benefit in various tumor types, including lung, colon, and breast cancers.^[^ ^34^, ^35^, ^36^ ^]^ CD8^+^ cytotoxic T cells are central to cancer immune surveillance. Infiltration of CD8^+^ T cells within the TME has been associated with a positive prognostic marker and may predict the response to ICB in certain types of cancers.^[^ ^9^, ^37^, ^38^ ^]^ However, in some cancer types, especially RCCs, higher levels of CD8^+^ T cells infiltration do not correlate with the survival benefit.^[^ ^39^ ^]^ Furthermore, a recently published proteogenomic analysis of ccRCC has found that the GP1 subtype of RCC, which has the strongest immune characteristics, is associated with the worst clinical outcome.^[^ ^12^ ^]^ A previous study has suggested that RCC utilizes T cell dysfunction strategy, which impairs the ability of cytotoxic T cells to kill tumor cells, rather than T cell exclusion strategy, for immune evasion.^[^ ^40^ ^]^ T cell dysfunction affects the response to ICB therapy. Although early‐stage dysfunctional T cells can be revived by anti‐PD‐1 therapy, dysfunctional T cells in late‐stage are resistant to ICB treatment.^[^ ^41^, ^42^ ^]^ Thus far, the molecular mechanisms underlying immune suppression and immune escape within the RCC microenvironment remain unclear. Our previous study established a five‐CpG‐based classifier for ccRCC prognosis, which identified EHBP1L1 as a prognosis‐related gene in RCC.^[^ ^25^ ^]^ In the present study, we identified EHBP1L1 as a crucial contributor to tumor immune evasion in RCC.

Currently, little is known about EHBP1L1, especially its function in RCC. The present study demonstrated that the expression level of EHBP1L1 was elevated in RCC at both the protein and mRNA levels. EHBP1L1 expression was associated with immune dysfunction and poor prognosis in TCGA‐KIRC cohort, which was further confirmed by our own RCC patient cohort. More importantly, by employing an immunocompetent murine RCC model and an immunodeficient nude mice model, we found that EHBP1L1 depletion significantly inhibited tumor growth and prolonged survival, which was mainly due to the enhanced antitumor immune response. Our high‐resolution scRNA‐seq further revealed that EHBP1L1 expressed in RCC cells inhibited intratumoral CD8^+^ T cells effector function, driving them toward a dysfunctional state. T cells cocultured with EHBP1L1‐deficient primary kidney tumor cells exhibited increased cytotoxicity of CD8^+^ T cells and more powerful antitumor function. Together, these results demonstrated the immunosuppressive role of EHBP1L1 in RCC, which may function through upregulating PD‐L1.

**Figure 8:**
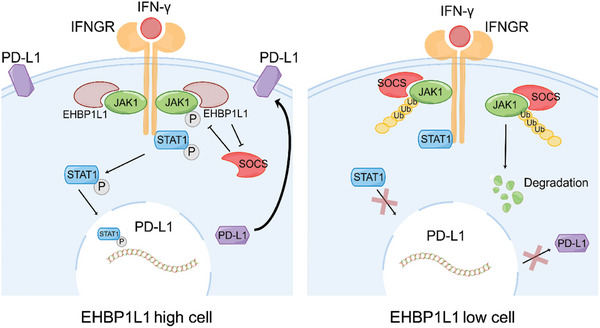
Schematic model depicting the key findings of this study. EHBP1L1 binds to the kinase domain of JAK1 and competes with SOCS1 to protect JAK1 from SOCS1‐mediated ubiquitination and degradation. In the absence of EHBP1L1, JAK1 is degraded, which impairs IFN‐*γ*/JAK1/STAT1 signaling and reduces PD‐L1 transcription.

The PD‐1/PD‐L1 axis is the main rate‐limiting step of the antitumor immune response. Blocking PD‐1/PD‐L1 signaling has exhibited potent antitumor activities and may obtain a long‐lasting response in various cancers, such as melanoma, non‐small cell lung cancer (NSCLC), RCC, and all microsatellite instability (MSI)‐high cancers.^[^ ^43^, ^44^, ^45^, ^46^ ^]^ However, the majority of patients do not benefit from ICB therapy, which may be attributed to medium‐high PD‐L1 expression, limiting the revival of antitumor immunity.^[^ ^47^ ^]^ The molecular mechanism that drives PD‐L1 overexpression remains to be explored. TFEB has been reported to enhance PD‐L1 expression in RCC cells, thus mediating resistance to mTOR inhibition.^[^ ^48^ ^]^ IFN‐*γ*, secreted by activated T cells and NK cells, stimulates tumor cells to overexpress PD‐L1 through the JAK‐STAT pathway.^[^ ^49^ ^]^ During tumor progression, IFN‐*γ*‐derived PD‐L1 promotes tumor immune evasion. IFN‐*γ* binds to type II interferon receptor and transactivates JAKs (JAK1 and JAK2), thereby activating the JAK‐STAT signaling pathway (mainly through STAT1). Phosphorylated STAT1, along with its downstream component (interferon‐responsive factor‐1, IRF‐1), binds to the PD‐L1 promoter and transcriptionally activates the expression of PD‐L1.^[^ ^16^ ^]^ Consistently, we found that EHBP1L1 upregulated JAK1‐STAT1 signaling to promote PD‐L1 expression. Interestingly, we identified JAK1 as an interacting protein of EHBP1L1. Ubiquitylation and degradation of JAK1 is a well‐known mechanism to regulate the host immune response. Ndfip1 and Ndfip2, activators of Nedd4 family ligases, have been reported to mediate the degradation of Jak1, thereby impairing T‐cell function.^[^ ^50^ ^]^ Similarly, ubiquitin ligases RNF125 and STUB1 bound to and ubiquitinated JAK1, resulting in JAK1 degradation and attenuated RTK expression.^[^ ^51^, ^52^ ^]^ In virology, influenza A virus polymerase protein PB2 and foot‐and‐mouth disease virus structural protein VP3, could also target and degrade JAK1 respectively, inhibiting IFN‐*γ*/JAK1/STAT signal transduction pathways.^[^ ^53^, ^54^ ^]^ In our study, we found that EHBP1L1 specifically interacts with JAK1 and blocks its interaction with SOCS1, which inhibits SOCS1‐mediated JAK1 ubiquitination and subsequent degradation. EHBP1L1 stabilizes JAK1, thereby promoting IFN‐*γ*/JAK1/STAT1 signaling and its downstream PD‐L1 expression (**Figure** **8**). Taken together, these findings elucidated a potential molecular mechanism of JAK1/STAT1 signaling overactivation and immunosuppressive TME in RCC.

In the TME, the IFN‐*γ*/STAT1 signaling pathway has divergent effects. Although the IFN‐*γ*/STAT1 signaling pathway enhances T cell infiltration and activation, thus forming an immunogenic TME,^[^ ^22^ ^]^ it also upregulates the expression of PD‐L1 and leads to immune escape and resistance to ICB.^[^ ^55^ ^]^ It has been reported that persistent IFN‐*γ* signaling allows tumors to acquire resistance to immunotherapy and that combined JAK/STAT and PD‐1/PD‐L1 inhibition has a synergistic effect compared to ICB monotherapy.^[^ ^46^ ^]^ In ccRCC, high IFN‐*γ*/STAT1 activity leads to high CD8^+^ T cell infiltration and immunosuppression scores, and it is associated with poor survival.^[^ ^12^ ^]^ In the present study, patients with low EHBP1L1 expression benefitted most from CD8^+^ T cells infiltration in TCGA‐KIRC cohort. Further analysis of patients from the Miao2018 cohort who received ICB therapy demonstrated that patients with low EHBP1L1 expression were more sensitive to ICB therapy. Furthermore, based on a preclinical PDX model with tumor‐specific CD8^+^ T cell transfer, we confirmed the synergistic effect of the combination of EHBP1L1 inhibition and PD‐1 blockade compared to PD‐1 blockade monotherapy. Inhibition of EHBP1L1 reprogrammed the immunosuppressive TME and sensitized tumors to PD‐1 blockade. Collectively, these results suggested that EHBP1L1 may be an attractive therapeutic target for the development of novel cancer therapeutics combined with ICB.

In summary, our study showed that EHBP1L1 mediates RCC immune escape by preventing JAK1 from ubiquitin‐dependent degradation, thus upregulating the IFN‐*γ*/JAK1/STAT1 signaling pathway and the transcription of PD‐L1. The combination of EHBP1L1 inhibition and ICB, which reprograms the immunosuppressive TME and prevents immune evasion, represents a potential therapeutic strategy for improving the efficacy of cancer immunotherapy in RCC patients.

## 4 Experimental Section

### Patients and Specimens

All human RCC tissue specimens were obtained from the Department of Urology, the First Affiliated Hospital, Sun Yat‐sen University (Guangzhou, China). The protocol was approved by the Medical Ethics Committee of the First Affiliated Hospital, Sun Yat‐sen University. RCC tissues treated with RNAlater and stored at ‐20 °C were used for qRT‐PCR analyses. RCC tissues stored at ‐80 °C were used for western blotting analyses.

### Cell Culture

The immortalized renal epithelial cell line (HK‐2), the human RCC cell lines (786‐O, 769‐P, A‐498, ACHN and Caki‐1) and the mouse RCC cell line (Renca) were purchased from American Type Culture Collection (ATCC). The human embryonic kidney 293T cell line, the human RCC OSRC2 cell line and other tumor cell lines (HCT‐116, A549, MAD‐MB231, AGS, SK‐Mel‐28, HeLa and Huh7 were purchased from the Cell Bank of the Chinese Academy of Sciences (Shanghai, China). The HK‐2 cell line was cultured in Keratinocyte Serum‐free Medium (SFM). The 293T and ACHN cell lines were cultured in Dulbecco's modified Eagle medium (DMEM). The Caki‐1 cell line was cultured in McCoy's 5A Medium. The remaining RCC cell lines were cultured in RPMI‐1640 medium. Other tumor cell lines were cultured as suggested by ATCC. All media were obtained from Gibco (USA) and supplemented with 1% penicillin‐streptomycin (Bioyard Biotechnology, China) and 10% fetal bovine serum (FBS) (BioChannel, China). All cell lines were examined with short tandem repeat (STR) profiling by the vendors and routinely tested for mycoplasma infection.

### Isolation of Primary RCC Cells

Tumor tissues, which were pathologically diagnosed as ccRCC, were isolated and washed with cold PBS (supplemented with 2% penicillin–streptomycin solution). The tumors were then cut into pieces and digested in DMEM containing 0.002% DNase I (Stemcell, Canada), 0.01% hyaluronidase (Stemcell, Canada), 0.2% collagenase IV (Stemcell, Canada) and 3 × 10^‐3^ m CaCl_2_ (21115, Sigma‐Aldrich) at 37 °C for 60 min with continuous shaking at 200 rpm. The digestion was centrifuged at 300 *g*, and the supernatant was filtered through a 40 µm cell strainer (352340, Corning). The cell suspension was cultured in a six‐well plate with DMEM containing 10% FBS and 1% penicillin–streptomycin solution.

### Antibodies and Reagents

The following primary antibodies were utilized in the western blot and immunoprecipitation analyses: JAK1 (3344, Cell Signaling Technology, CST), p‐JAK1 (74129, CST), JAK2 (3230, CST), p‐JAK2 (3771, CST), STAT1 (14994, CST), p‐STAT1 (9167, CST), STAT3 (9139, CST), p‐STAT3 (9145, CST), anti‐human EHBP1L1 (Abcam, ab122557), anti‐mouse EHBP1L1 (orb183327, Biorbyt), SOCS1 (3950, CST), Flag tag (AE005, ABclonol), His tag (AE003, ABclonol), mouse control IgG (AC011, ABclonol), rabbit control IgG (AC005, ABclonol), PD‐L1 (66248‐1‐Ig, Proteintech) and GAPDH (60004‐1‐Ig, Proteintech). The following antibodies were used for IHC staining: anti‐human EHBP1L1 (Abcam, ab122557), JAK1 (3344, CST) and PD‐L1 (66248‐1‐Ig, Proteintech). The secondary antibodies used in the immunofluorescence assay were as follows: CoraLite488 (SA00013‐2, Proteintech) and CoraLite594 (SA00013‐3, Proteintech). The following antibodies were used for flow cytometry analyses: FITC anti‐human CD8a (300905, Biolegend), PE anti‐mouse CD8a (162303, Biolegend), FITC anti‐mouse CD8a (100705, Biolegend), PE anti‐mouse TNF‐*α* (506305, Biolegend), PE anti‐human TNF‐*α* (376203, Biolegend), PE anti‐mouse Perforin (154305, Biolegend), PerCP/Cyanine5.5 anti‐human Perforin (308113, Biolegend), FITC anti‐human/mouse Granzyme B (372205, Biolegend), PE anti‐human/mouse Granzyme B (372207, Biolegend), PE anti‐mouse CD45 (147711, Biolegend), FITC anti‐mouse CD4 (100405, Biolegend), Alexa Fluor 700 anti‐mouse FOXP3 (126421, Biolegend), APC anti‐mouse CD19 (152409, Biolegend), APC anti‐mouse F4/80 (123115, Biolegend), FITC anti‐mouse/human CD11b (101205, Biolegend), PE anti‐mouse IFN‐*γ* (505807, Biolegend), PE anti‐human IFN‐*γ* (502508, Biolegend), APC anti‐human CD3 (317317, Biolegend), APC anti‐mouse CD3 (100235, Biolegend), FITC anti‐human PD‐L1 (393605, Biolegend) and FITC anti‐human HLA‐A2 (343303, Biolegend). MG‐132 (HY‐13259) and CHX (HY12320) were purchased from MedChemExpress.

### Plasmid, RNA Interference and Lentivirus Construction

Flag‐tagged EHBP1L1, Flag‐tagged full length JAK1, Flag‐tagged truncated JAK1, and His‐tagged ubiquitin were cloned into the pcDNA3.1(+) vector (Genecopoeia, China). All the plasmids were sequenced before transfection to confirm the correct nucleotide sequence. Small interfering RNAs targeting EHBP1L1 and SOCS1 for in vitro and in vivo experiments were synthesized by RiboBio (China). The shRNA lentivirus and overexpression lentivirus were constructed, identified, and provided by Bioyard Biotechnology (China). The targeted sequences are shown in Table S3 (Supporting Information).

### Primary Human Peripheral Blood Mononuclear Cell (PBMC) and CD8^+^ T Cell Isolation

PBMCs were isolated from healthy donors using Ficoll‐Paque (17‐5442‐02, GE Healthcare) density gradient centrifugation following the manufacturer's instructions, and PBMCs were washed twice with PBS. Primary human CD8^+^ T cells were purified using a CD8^+^ T cell Isolation Kit (Miltenyi, 130‐096‐495) following the manufacturer's instructions. CD8^+^ T cells were confirmed to be > 95% pure by flow cytometric analysis.

### Quantitative Real‐Time PCR (qRT‐PCR) Analyses

Total RNA from tumor tissues or cell lines was extracted with TRIzol (Invitrogen). Total RNA was reverse transcribed using 4×Reverse Transcription Master Mix (EZBioscience, USA) according to the manufacturer's instructions. Quantitative real‐time PCR was performed using 2×SYBR Green qPCR Master Mix (EZBioscience, USA) and a Roche LightCycler 480 Instrument. The forward and reverse primers used in this study are listed in Table S4 (Supporting Information).

### Western Blotting Analysis

Cells were lysed in cell lysis buffer (Beyotime, China) with protease inhibitor cocktail (CoWin Biosciences, China) on ice and then collected with cell scrapers (BIOFIL, China). Protein quantitation was performed with Pierce BCA Protein Assay Kit (ThermoFisher, USA) and measured at a wavelength of 562 nm (MD VersaMax, USA). Protein samples were loaded in 7.5%–12.5% SDS‐PAGE gels. After electrophoresis, proteins were transferred to PVDF membranes (Merck Millipore, USA) in an electrophoretic transfer unit (Tanon, China). After blocking, the PVDF membranes were incubated with primary antibodies at 4 °C for more than 12 h. After 1 h of incubation with secondary antibodies at room temperature, the protein bands were detected by chemiluminescence (Tanon).

### Co‐IP Assay and LC‐MS/MS Analysis

Cells transfected with the indicated plasmids were lysed in cell lysis buffer (Beyotime) containing protease inhibitor cocktail (CoWin Biosciences). Then, 10% of the input was collected and stored at ‐20 °C until further analysis. Protein samples were incubated with protein A/G magnetic beads (Thermo Scientific) and antibodies at 4 °C overnight. After washing the magnetic beads five times for 5 min, the supernatant was removed. Finally, 1 × SDS loading buffer was added to the magnetic beads for protein denaturation. IP samples were stored at ‐20 °C or directly used for electrophoresis, silver staining or LC‐MS/MS analysis (Wininnovate Bio, China). The protein identification and quantification were performed using Thermo Scientific Proteome Discovery Software version 1.4.

### Immunofluorescence (IF) Staining

Cells were cultured in confocal dishes for 24 h. Cells were then fixed with 4% paraformaldehyde (Beyotime) for 15 min at room temperature and permeabilized with 0.5% TritonX‐100 for 15 min at room temperature. After blocking with 5% BSA (Sigma‐Aldrich) for 1 h at room temperature, cells were incubated with anti‐EHBP1L1 antibody (orb183327, Biorbyt) or anti‐JAK1 antibody (66466‐1‐Ig, Proteintech) overnight at 4 °C. Cells were then incubated with the following secondary antibodies for 1 h at room temperature: CoraLite488 (SA00013‐2, Proteintech) or CoraLite594 (SA00013‐3, Proteintech). Images were acquired using an OLYMPUS FV1000 confocal microscopy (Japan).

### Immunohistochemistry (IHC)

For IHC staining, paraffin‐embedded tissues were deparaffinized and rehydrated, and they were then subjected to antigen retrieval (ZLI‐9079, ZSGB‐BIO, China), endogenous peroxidases blocking (PV‐6001, ZSGB‐BIO), blocking (ZLI‐9056, ZSGB‐BIO), antibody incubation and staining (ZLI‐9017, ZSGB‐BIO). The staining index (SI) was evaluated by two independent pathologists. The staining intensity was defined as follows: negative = 0, weak = 1, intermediate = 2, and strong = 3. The proportion of positive cells was defined as follows: <5% = 0, 5–25% = 1, 26–50% = 2, 51–75% = 3, and 76–100% = 4. The SI was calculated as follows: SI = (staining intensity) (0‐3) × (proportion of positive cells) (0‐4).

### Flow Cytometry

Tumor cell digestion was performed as previously described for the isolation of primary RCC cells. After filtering, tumor cells were resuspended in PBS containing 5 × 10^‐3^ m EDTA and 1% FBS. For measurement of PD‐L1 expression, non‐immune cells were removed via density gradient centrifugation, and the remaining cells were stained with anti‐PD‐L1 (66248‐1‐Ig, Proteintech) or isotype‐control antibodies. After three washes with PBS, cells were stained with Alexa Fluor 488‐conjugated goat anti‐mouse IgG (A‐11001, Invitrogen) for 20 min. For detection of cytotoxic cytokines production, cells were treated with 50 ng mL^‐1^ phorbol 12‐myristate 13‐acetate (PMA), 1 × 10^‐6^ m ionomycin and protein transport inhibitor (BD) for 6 h at 37 °C. Cells were then fixed and permeabilized with a Fixation and Permeabilization Solution Kit (BD, 554714) following the manufacturer's instructions, and cells were then stained with the indicated primary antibodies. For measurement of HLA‐A2 expression, tumor cells were stained with FITC anti‐human HLA‐A2 (343303, Biolegend) or isotype‐control antibodies. For apoptosis analysis, cells were collected and evaluated by the Annexin V‐APC/PI apoptosis kit (AP‐107, Multi Science) according to the manufacturer's instructions.

Samples were analyzed with a Beckman CytoFLEX Flow cytometer (Beckman Coulter, USA), and FlowJo10 software was used to analyze the data.

### RNA‐Seq Analysis

Total RNA was extracted from shCtrl and shEHBP1L1 786‐O cells using TRIzol (Invitrogen) according to the manufacturer's instructions. The mRNA was enriched by removing ribosomal RNA, digested and reverse‐transcribed into second‐strand cDNA. The cDNA library construction and sequencing were performed by Tsingke Biotechnology (China). The high‐quality raw sequencing reads were mapped to the human reference genome (GRCh38) using the Hisat2 alignment tool. The gene expression level was normalized by fragments per kilobase of transcript per million mapped reads (FPKM). DESeq2 was used to identify differentially expressed genes (DEGs).

### Generation of DCs and Tumor‐Specific CD8^+^ T Cells

For the generation of DCs, mononuclear cells were obtained from the peripheral blood of HLA‐A2^+^ healthy donors and cultured in VIVO medium (04‐418Q, Lonza) supplemented with 100 ng mL^‐1^ GM‐CSF and 30 ng mL^‐1^ IL‐4 (PeproTech, USA). The medium and cytokines were replaced every 3 d. At Day 6, DCs were mature, and they were stimulated with 10 ng mL^‐1^ TNF‐*α* (PeproTech) for 24 h. The DCs were then pulsed for another 24 h with tumor lysates from HLA‐A2^+^ patients by freeze‐thawing with liquid nitrogen. To generate tumor‐specific CD8^+^ T cells, CD8^+^ T cells were isolated from the peripheral blood of the same donors as described above. The isolated CD8^+^ T cells were cocultured with mature DCs at a ratio of 5:1 in VIVO medium (Lonza, Switzerland) containing 25 IU mL^‐1^ IL‐2 (PeproTech) for 6 d to induce tumor‐specific T cells.

### Cytotoxicity Assays

Tumor‐specific CD8^+^ T cells were generated as described above and cocultured with HLA‐A2^+^ primary kidney tumor cells at an effector/target (E/T) ratio of 10:1 in 48‐well plates for 12 h at 37 °C. Tumor cells were then stained with PI (ST511, Beyotime) and immediately analyzed by flow cytometry. T cells were collected and then treated with protein transport inhibitor (BD) for 6 h at 37 °C followed by fixation and permeabilization using a Fixation and Permeabilization Solution Kit (554714, BD) according to the manufacturer's instructions. Cells were then stained with PE anti‐human IFN‐*γ* antibody (502508, Biolegend). Samples were analyzed with a Beckman CytoFLEX Flow cytometer (Beckman Coulter, USA), and FlowJo10 software was used to analyze the data.

### In Vivo Mouse Experiments

The in vivo mouse experiments were approved by the Institutional Animal Care and Use of Sun Yat‐sen University Cancer Center (SYSUCC) and performed in accordance with the guidelines for the care and use of animals. Six‐ to eight‐week‐old male BALB/c mice, BALB/‐Nu mice or NCG mice (NOD/ShiLtJGpt‐Prkdcem26Cd52Il2rgem26Cd22/Gpt) were purchased from GemPharmatech (China) and fed in standard pathogen‐free (SPF) conditions. BALB/c mice and BALB/‐Nu mice were subcutaneously injected with stably transfected Renca cells (5 × 10^5^ cells/100 µL). The palpable tumor weight was measured every day. In some experiments, to deplete CD8^+^ or CD4^+^ T cells in vivo, CD8‐depleting antibody (BioXcell, BP0117) (200 µg per mice in 100 µL PBS) or CD4‐depleting antibody (BioXcell, BP0003‐1) (200 µg per mice in 100 µL PBS) were intraperitoneally injected 1 day before tumor injection, followed by three consecutive injections every 3 d. Depletion efficiency was checked by flow cytometry with spleen on the day sacrificing the mice using antibodies targeting non‐competing CD8 epitopes or CD4 epitopes.

For the PDX models, fragments of fresh human RCC tumors were subcutaneously transplanted into NCG mice. When the tumor volume reached 100 mm^3^, the NCG mice were sacrificed. The tumors were separated and cut into 1 mm^3^ pieces. Tumor pieces were transplanted into the next‐generation NCG mice. After three passages, a stable RCC PDX model was successfully established. When the tumor volume reached 100 mm^3^, NCG mice were randomly divided into the following five groups: control group, TIL treatment group, TIL + siEHBP1L1 treatment group, TIL + *α*PD‐1 treatment group, and TIL + siEHBP1L1 + *α*PD‐1 treatment group. Human DCs and tumor‐specific CD8^+^ T cells were generated as described above. For adoptive T cell transfer, tumor‐specific CD8^+^ T cells (2.5 × 10^6^ cells per mouse) and DC cells (0.5 × 10^6^ cells per mouse) were injected via tail vein to rebuild the human immune system. Mice were then administered EHBP1L1‐siRNA (siEHBP1L1) or negative siRNA control (RiboBio, China) via intratumor injection (5 nmol every 4 d for a total of six times). For anti‐PD‐1 treatment, mice were intraperitoneally injected with anti‐PD‐1 antibody (BioXcell, USA) (100 µg per mouse every 4 d for a total of seven times). The palpable tumor weight was measured every week. The tumor volume (mm^3^) was calculated as follows: tumor volume = (length × width^2^) / 2. The mice were sacrificed when the tumor size reached 1500 mm^3^ or ulceration occurred. The tumors were separated surgically for IHC staining.

For the orthotopic xenograft models, 6 to 8 week old male BALB/c mice and BALB/‐Nu mice were anesthetized with 1% pentobarbital (50 mg kg^‐1^) by intraperitoneal injection. Stably transfected Renca cells (5 × 10^5^ cells/25 µL) were orthotopically injected into the subcapsular of the left kidney. The survival of mice was recorded.

### Single‐Cell RNA Sequencing

Six to eight week old male BALB/c mice were inoculated subcutaneously with shCtrl or shEHBP1L1 Renca cells (5×10^5^ cells/100 µL, *n* = 2 per group). Fresh mouse tumor tissues were collected 14 days post inoculation and single‐cell suspensions were prepared after mechanical disruption and enzymatic digestion with 1 mg mL^‐1^ Type IV Collagenase (Sigma, USA). After staining, tumor‐infiltrating CD8^+^ T cells (CD3^+^ CD8^+^) were sorted by a BD FACS Aria Cell Sorter and immediately processed for scRNA‐seq. FACS‐sorted CD8^+^ T cells were processed and libraries were constructed according to the Chromium Single Cell 3′ Library V3 Kit (10x Genomics, USA) following the recommended protocol and sequenced on Novaseq 6000 (llumina, USA).

### Single‐Cell Sequencing Data Processing

The feature‐barcode unique molecular identifier (UMI) matrices were generated from sequencing data by Cell Ranger (version 6.0.2, 10× Genomics) Pipeline. The murine reference genome mm10 was used to map reads and quantify expression levels. The output filtered gene expression matrices were further analyzed by the Seurat R package (v 4.0). Genes expressed at a proportion > 0.1% of the data and cells with > 300 genes detected were selected for further analyses. Cells that expressed more than 10% UMIs derived from the mitochondrial genome were excluded. After removal of low‐quality cells, the gene expression matrices were normalized and scaled for each gene across all cells and then integrated, scaled, and clustered on the low‐dimensional space with the *RunUMAP* function with default settings. “AddModuleScore” function of Seurat package was performed to calculate the mean expression of genes in certain gene sets. Eight marker genes for CD8^+^ effector T cell (*Nkg7*, *Gzma*, *Gzmb*, *Ifng*, *Ccl4*, *Cst7*, *Prf1* and *Ccl3*) and seven inhibitory marker genes for CD8^+^ exhausted T cell (*Pdcd1*, *Ctla4*, *Havcr2*, *Lag3*, *Tigit*, *Tox* and *Layn*) were applied to calculate the cytotoxic score and exhaustion score, respectively. For trajectory analysis, the R package “monocle” was applied to conduct pseudotime ordering and infer the state evolution process of CD8^+^ T cells. All details regarding the Seurat analyses performed in this work can be found in the website tutorial (https://satijalab.org/seurat/v3.0/pbmc3k_tutorial.html).

### Enzyme‐Linked Immunosorbent Assay

Blood samples were collected using a serum separator tube from mice before euthanasia. Then, samples were allowed to clot at room temperature for 1 h followed by centrifugation for 10 min at 3000 × *g*. Serum was then transferred to a new tube and stored at ‐80 °C before analysis. For coculture assays, supernatant from shCtrl or shEHBP1L1 primary kidney tumor cells co‐cultured with tumor‐specific CD8^+^ T cells was collected and stored at ‐80 °C. IFN‐γ and TNF‐*α* levels in the serum samples were measured with a human IFN‐γ and TNF‐*α* ELISA kit (Elikine, USA).

### Bioinformatics Analysis

The clinical data and RNA‐seq data of TCGA‐KIRC cohort were downloaded from Firebrowse (http://firebrowse.org/). The clinical data and proteomic data, including 110 primary tumors and 84 normal tissues, of ccRCC patients were downloaded from UALCAN (http://ualcan.path.uab.edu/) and CPTAC (https://pdc.cancer.gov/pdc/browse). The tumor immune infiltration analyses were performed using the CIBERSORT algorithm (https://cibersortx.stanford.edu/). Gene set enrichment analysis (GSEA) was performed using GSEA software version 4.1.0 (Broad Institute, USA). T cell dysfunction analyses were performed using TIDE tools (http://tide.dfci.harvard.edu/).

Relative cytotoxicity level was assessed as described previously.^[^ ^56^ ^]^ Briefly, a deconvolution method, ICTD (Inference of cell types and deconvolution), was utilized to estimate the relative proportion of total T cells in TCGA‐KIRC samples using the expression profile of *CD2*, *CD3D*, *CD3E*, *CD3G*, and *CD8A*. Then, the expression profile of *CCL5*, *CD3D*, *GZMA*, *GZMB*, *IFNG*, and *NKG7* was utilized to estimate the whole tissue cytotoxicity level in the samples. Relative cytotoxicity level was computed by relative cytotoxicity level = (whole tissue cytotoxicity level)/(total T cell proportion). Normalized expression levels of 3 cytotoxicity markers in KIRC samples were computed by normalized gene expression = (gene expression level)/(total T cell proportion).

### Statistical Analyses

Statistical analyses were performed using SPSS version 22.0 or GraphPad Prism 9 software. Data are presented as the mean ± standard deviation (SD). All in vitro experiments were performed with at least three independent biological replicates. Data were analyzed for normality before comparisons. For comparisons between two groups, statistical significance was determined by two‐tailed Student's t test. For multiple comparisons, one‐way ANOVA with Tukey's post hoc test was used. For the survival analyses, OS was defined as the time from the operation to the date of death for any reason. Kaplan‐Meier survival curves were plotted with log‐rank tests. Correlation analyses were performed using Pearson's correlation (continuous variables) or Spearman's correlation (discontinuous variables). A *P* value less than 0.05 was considered significant. Statistical significance was shown as *(*P* < 0.05), ** (*P* < 0.01) or *(*P* < 0.001).

## Conflict of Interest

The authors declare no conflict of interest.

## Supporting information

Supporting Information

## Data Availability

The data that support the findings of this study are available from the corresponding author upon reasonable request.
