# Supplementary material for: EHBP1L1 Drives Immune Evasion in Renal Cell Carcinoma through Binding and Stabilizing JAK1
Source: Adv Sci (Weinh). 2023 Feb 12;10(11):2206792. doi: 10.1002/advs.202206792 (PMC10104659; doi:10.1002/advs.202206792)
Supplement: Supplementary file 1 — Supporting Information [file ADVS-10-2206792-s001.pdf]

ADVANCED SCIENCE
Open Access

Supporting Information

for *Adv. Sci.*, DOI 10.1002/advs.202206792

EHBP1L1 Drives Immune Evasion in Renal Cell Carcinoma through Binding and Stabilizing JAK1

Yihui Pan, Guannan Shu, Liangmin Fu, Kangbo Huang, Xinwei Zhou, Chengpeng Gui, Huashan Liu, Xiaohan Jin, Minyu Chen, Pengju Li, Junjie Cen, Zihao Feng, Jun Lu, Zhenhua Chen, Jiaying Li, Quanhui Xu, Yinghan Wang, Hui Liang, Zhu Wang, Qiong Deng, Wei Chen, Junhang Luo, Jiefeng Yang*, Jiaxing Zhang* and Jinhuan Wei**

Supporting Information

EHBP1L1 drives immune evasion in renal cell carcinoma through binding and stabilizing JAK1

Yihui Pan, Guannan Shu, Liangmin Fu, Kangbo Huang, Xinwei Zhou, Chengpeng Gui, Huashan Liu, Xiaohan Jin, Minyu Chen, Pengju Li, Junjie Cen, Zihao Feng, Jun Lu, Zhenhua Chen, Jiaying Li,Quanhui Xu, Yinghan Wang, Hui Liang, Zhu Wang, Qiong Deng, Wei Chen , Junhang Luo, Jiefeng Yang, Jiaxing Zhang,* Jinhuan Wei**

Supplementary Figure 1

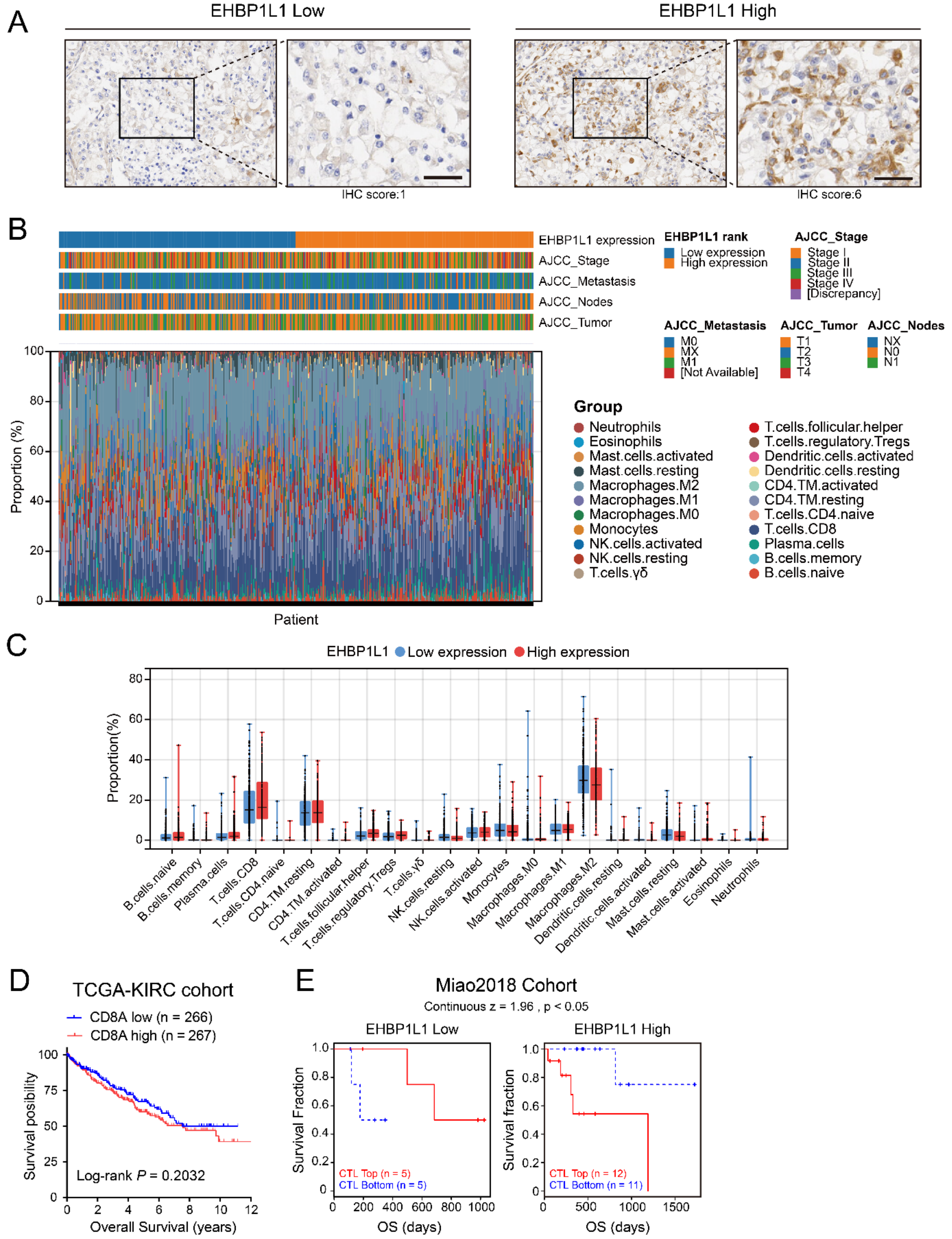

Supplementary Figure 1. EHBP1L1 is overexpressed and is associated with immunosuppression in RCC. A) Representative immunohistochemical images for low and high EHBP1L1 expression in human RCC tissues. Scale bar = 100 μm. **B)** EHBP1L1 expression group and proportions of TME cells for 533 patients in TCGA-KIRC cohort. Tumor stage, metastasis status, tumor grade and node metastasis status are shown as patient

annotations. **C)** Proportions of TME cells in different EHBP1L1 subgroups. The scattered dots represent the immune score of the two subgroups, and the thick lines represent the median value. **D)** Overall survival of RCC patients in TCGA-KIRC cohort, stratified by CD8A expression. **E)** Association among EHBP1L1 expression, cytotoxic T lymphocyte level (CTL) and OS for patients with metastatic ccRCC. The samples were divided according to EHBP1L1 expression to show the association between the CTL level and survival outcome. The left panel shows ccRCC patients with low EHBP1L1 expression, and the right panel shows ccRCC patients with high EHBP1L1 expression. All experiments were performed with three independent biological replicates.

Supplementary Figure 2

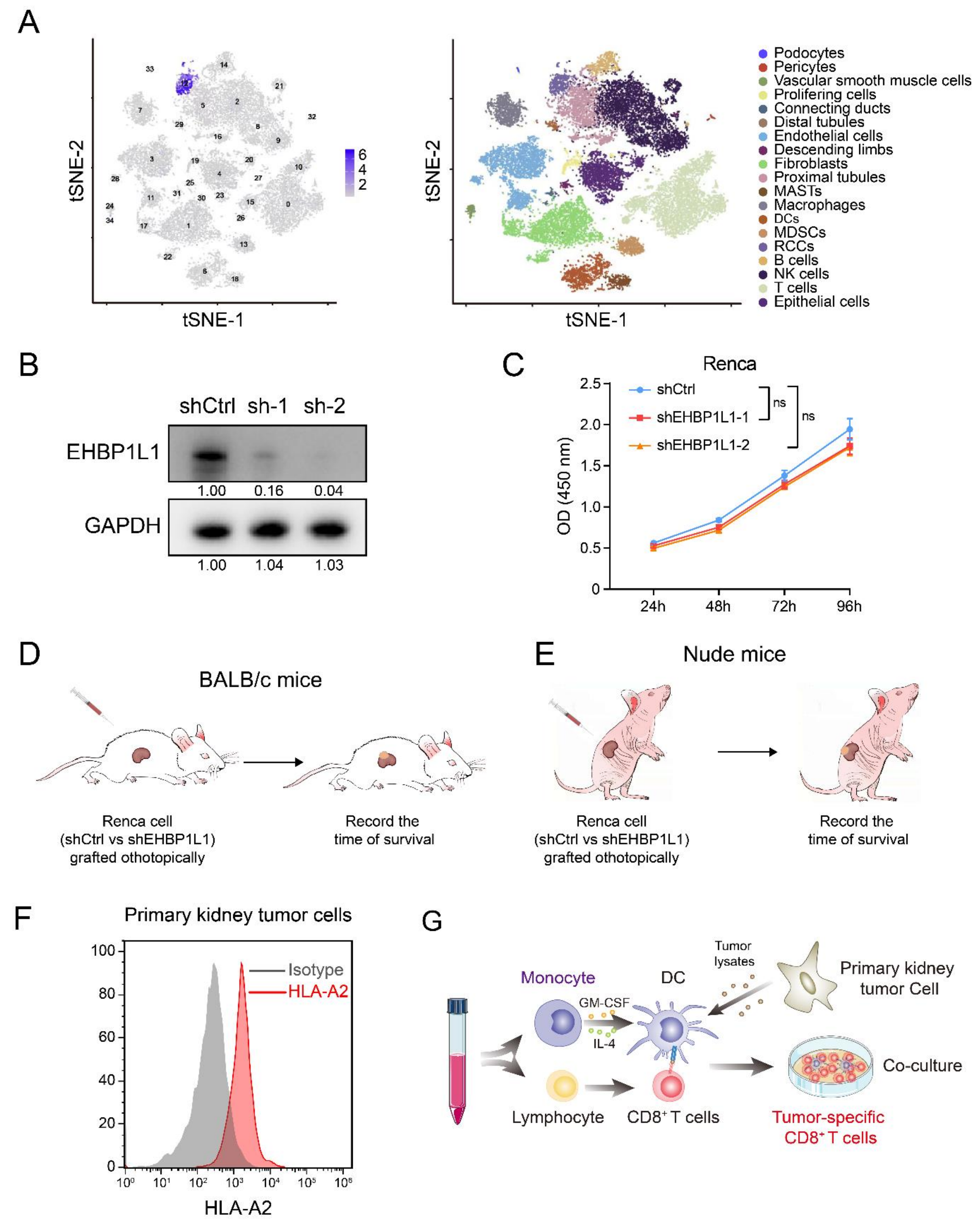

Supplementary Figure 2. EHBP1L1 silencing enhances antitumor immunity in murine and human RCC. A) t-SNE plot of RCC cell atlases (scRNA-seq database GSE159115) indicates the expression of EHBP1L1 in RCC tumor microenvironment. Immune cells, stromal cells, benign kidney cells and RCC cells are colored by cell types. Left panel, EHBP1L1 expression in different cell types; Right panel, identifier. **B)** Representative western

blot of EHBP1L1 protein expression in Renca cells with EHBP1L1 knockdown. **C)** CCK8 assays showing the proliferation of Renca cells with EHBP1L1 knockdown. **D, E)** Schematic protocols of Renca cells with or without EHBP1L1 knockdown orthotopically implanted into immunocompetent BALB/c (D) and immunodeficient nude (E) mice (n = 12 per group). **F)** Flow cytometry analysis of HLA-A2 expression in primary kidney tumor cells. **G)** Schematic protocol of inducing primary kidney tumor-antigen activated $CD8^+$ T cells. All experiments were performed with three independent biological replicates.

Supplementary Figure 3

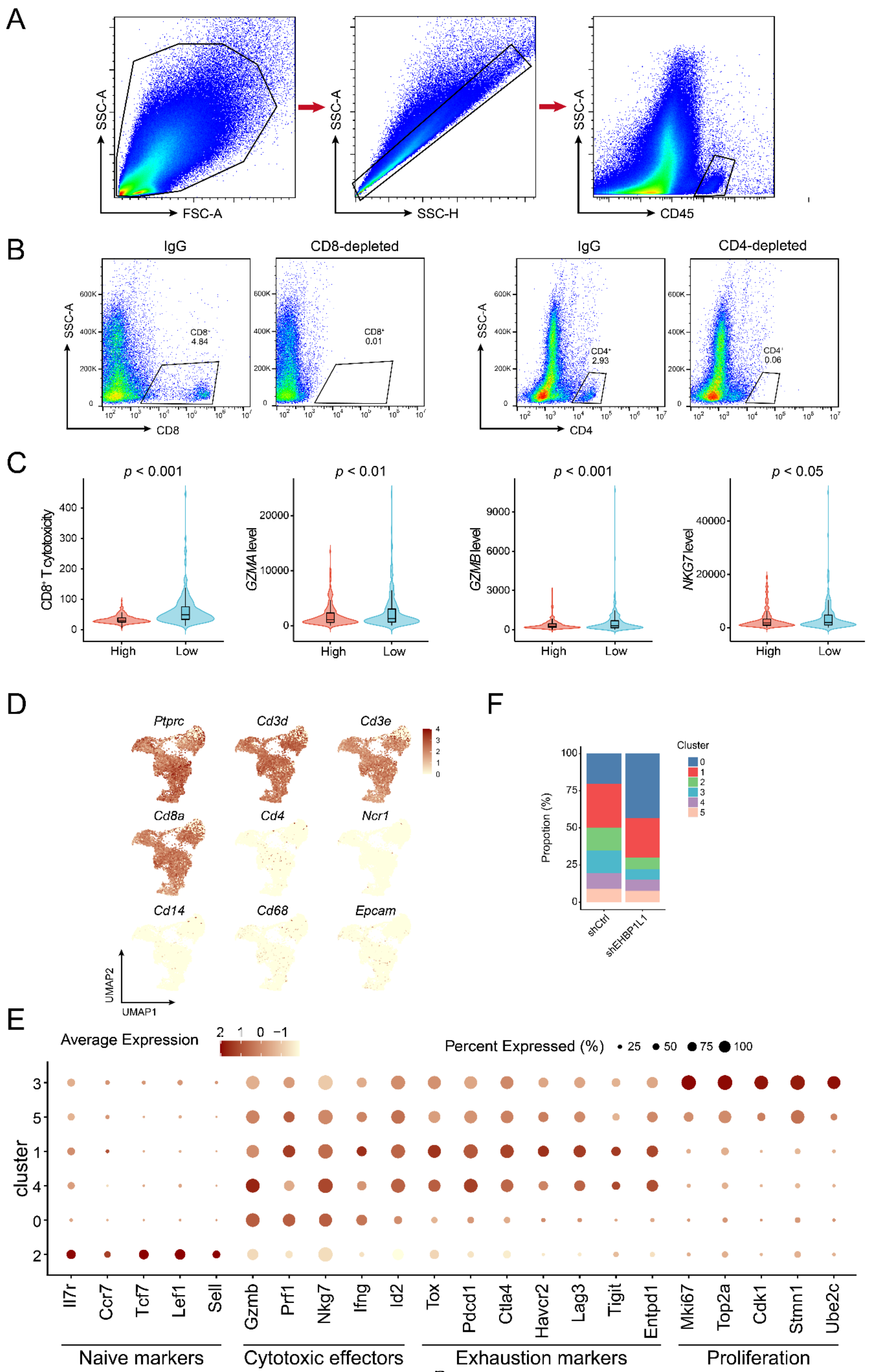

Supplementary Figure 3. Effect of RCC EHBP1L1 expression in CD8$^+$ T cells in the tumor microenvironment. **A)** Representative flow plots of enriching CD45$^+$ immune cells from Renca tumors for further analysis. **B)** Representative flow cytometry plots showing relative abundance of CD8$^+$ and CD4$^+$ T cells on the day sacrificing the mice with isotype control and depleting anti-CD8/CD4 antibody treatment. **C)** Violin plots showing relative Cytotoxicity level of tumor-infiltrating CD8$^+$ T cells and normalized expression levels of 3 cytotoxic markers (*GZMA*, *GZMB* and *NKG7*) in TCGA-KIRC cohort with high or low EHBP1L1 expression levels. **D)** Expression of indicated marker genes on UMAP plot. **E)** Dot plots showing the expression of indicated functional genes between CD8$^+$ T cell clusters. Dot size is proportional to the fraction of cells expressing indicated genes. Color intensity corresponds to the average expression levels of indicted genes. **F)** Percentage distribution of CD8$^+$ T cell clusters in shCtrl and shEHBP1L1 group. Each experiment was repeated three times with 5 mice per group, and data shown are representative of three independent experiments (A, B).

Supplementary Figure 4

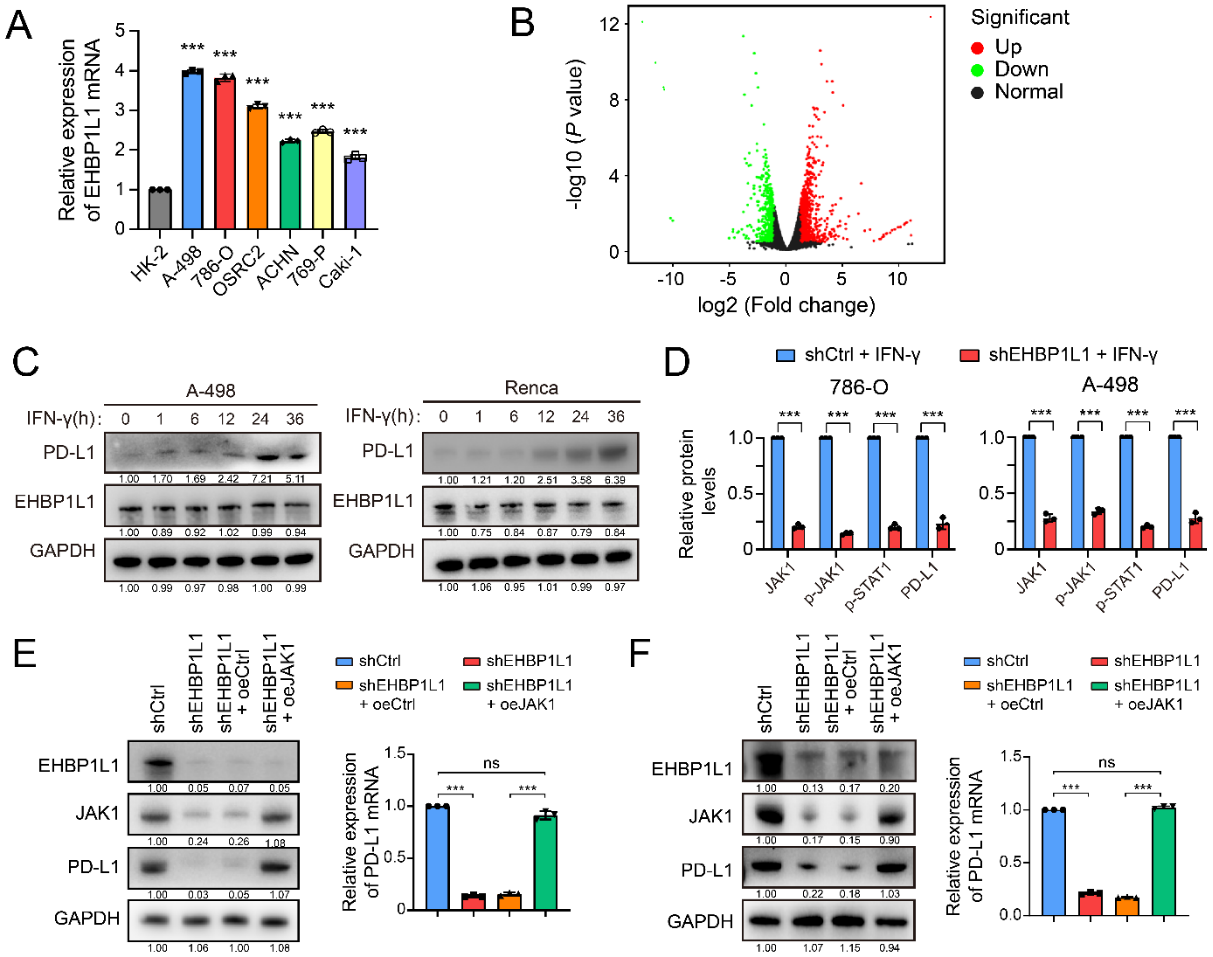

Supplementary Figure 4. EHBP1L1 knockdown reduces JAK1-STAT signaling activity in RCC. A) Relative mRNA expression of EHBP1L1 by qPCR in immortalized renal epithelial cell line HK-2 and RCC cell lines (A-498, 786-O, OSRC2, ACHN, 769-P and Caki-1) according to qPCR. GAPDH was used as a loading control9. **B)** Volcano plot of RNA-seq data for shCtrl and shEHBP1L1 786-O cells. **C)** Representative western blot showing PD-L1 protein expression levels after IFN-γ stimulation in A-498 cells (left panel) and Renca cells (right panel). Cells were treated with 100 U/ml IFN-γ for the indicated times. **D)** Statistical analysis of JAK1, p-JAK1, p-STAT1 and PD-L1 protein expression levels in 786-O (left panel) and A-498 (right panel) cells as shown in Figure 4G. **E)** Effects of JAK1 overexpression on PD-L1 expression in 786-O cells with EHBP1L1 knockdown. **F)** Effects of JAK1 overexpression on PD-L1 expression in Renca cells with EHBP1L1 knockdown. All experiments were performed with three independent biological replicates, and data shown are representative of three independent experiments.

Supplementary Figure 5

A

MS result of candidate EHBP1L1 interacting proteins

Protein	-10lgP	Peptide (#)	Coverage (%)
HS71B	180.81	13	22
BIP	161.88	13	23
ACTS	138.14	10	23
K2C1B	137.01	7	8
JAK1	132.63	9	9

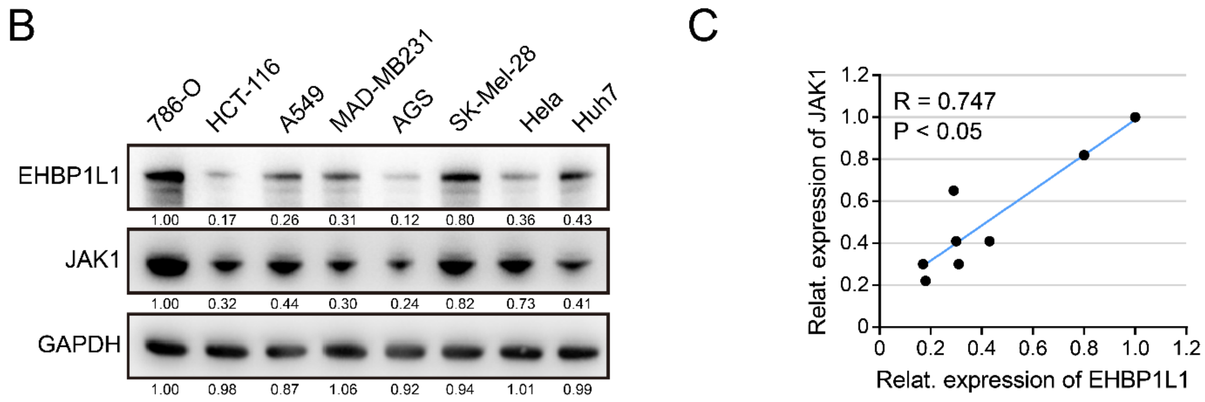

Supplementary Figure 5. EHBP1L1 interacts with JAK1. A) Proteins that specifically interact with EHBP1L1 in 786-O cells were identified by LC/MS. 786-O cells stably expressing Flag-EHBP1L1 were purified with Flag or control IgG antibody and analyzed by MS. The table lists the proteins that bind to Flag-EHBP1L1 but not IgG as identified by MS. **B, C)** Representative Western blot image (B) and correlation analysis (C) of expression of EHBP1L1 and JAK1 in a panel of tumor cells. All experiments were performed with three independent biological replicates, and data shown are representative of three independent experiments.

Supplementary Figure 6

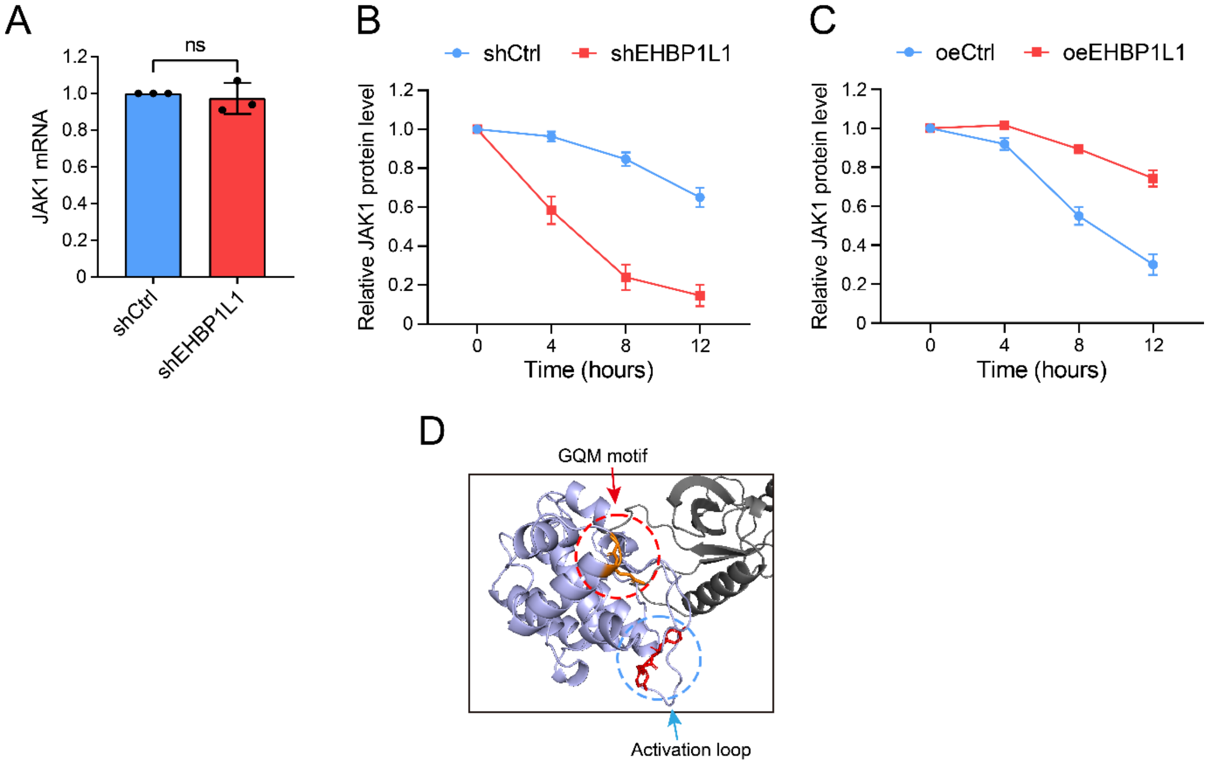

Supplementary Figure 6. Binding of EHBP1L1 to JAK1 increases its stability by competing with SOCS1. A) mRNA expression of JAK1 in 786-O cells with EHBP1L1 knockdown. **B, C**) Statistical analysis of JAK1 protein degradation after EHBP1L1 knockdown in 786-O cells (B) and EHBP1L1 overexpression in Caki-1 cells (C) as evaluated by CHX assay. **D)** The predicted structure of JAK1 by AlphaFold showing the GQM motif and JAK1 activation loop (Tyr 1034 and 1035) in the kinase domain. All experiments were performed with three independent biological replicates, and data shown are representative of three independent experiments.

Supplementary Figure 7

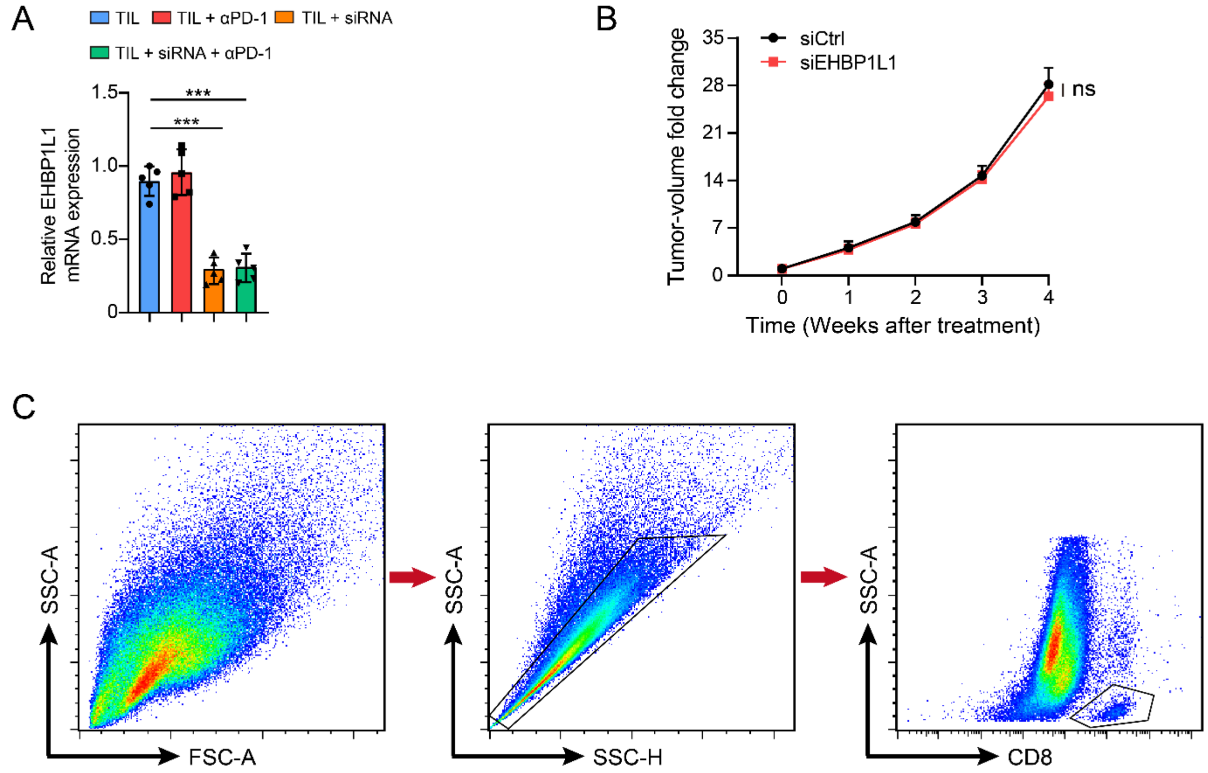

Supplementary Figure 7. EHBP1L1 siRNA enhances the efficacy of anti-PD-1 therapy in human RCC PDX models. A) qPCR analysis showing EHBP1L1 expression levels in tumor tissues from PDX mice with the indicated treatments (n = 5 per group). **B)** Tumor growth curves of RCC PDX tumor growth in NCG mouse without immune reconstitution treated with control or EHBP1L1 siRNA (n = 5 per group). **C)** Representative flow plots of enriching $CD8^{+}$ T cells from RCC PDX tumors for further analysis. Data represent one independent experiment with 5 mice per group (A, B). Each experiment was repeated three times with 5 mice per group, and data shown are representative of three independent experiments (C).

Supplementary Table 1. Correlations between EHBP1L1 expressions and clinical characteristics of ccRCC patients in the SYSU Cohort.

Parameters	Number of cases	EHBP1L1		*P* Value
		Low	high	
Total	120	60	60	
Age				0.551
≤60	85	41	44	
>60	35	19	16	
Gender				0.181
male	79	43	36	
female	41	17	24	
WHO/ISUP Grade				0.043
I-II	95	52	43	
III-IV	25	8	17	
TNM Stage				0.227
I-II	108	56	52	
III	12	4	8	

Supplementary Table 2. Univariate and multivariate cox regression analysis of variables associated with OS in the SYSU cohort.

Parameters	Univariate analysis		Multivariate analysis	
	**HR (95%CI)**	***P* value**	**HR (95%CI)**	***P* value**
Age (≥60 years vs. <60 years)	2.23(0.90-5.48)	0.082	1.96(0.78-4.93)	0.15
Gender (male vs. female)	0.56(0.23-1.38)	0.21	0.75(0.29-1.96)	0.56
WHO/ISUP Grade (I-II vs. III-IV)	2.93(1.09-7.86)	0.033	1.85(0.65-5.26)	0.25
TNM Stage (I-II vs. III)	3.52(1.16-10.71)	0.026	2.94(0.94-9.22)	0.065
EHBP1L1 expression (low vs. high)	3.83(1.37-10.69)	0.010	3.14(1.08-9.14)	0.035

HR=hazard ratio. CI= confidence interval

Supplementary Table 3. Targeted sequences of siRNA oligos and shRNAs used in this study.

Name	Targeted sequences
**human genes**	
si-EHBP1L1-1	CACCGATTCTACCCAGACA
si-EHBP1L1-2	CCAGGAAGTCACCACTGGCTA
si-SOCS1	TGCCGTGTTATTTTGTTATTACT
shEHBP1L1-1	atTTATTTGTCACCGAGGGTG
shEHBP1L1-2	CCAGGAAGTCACCACTGGCTA
**murine genes**	
si-EHBP1L1-1	ACCAGTATGAAACCAAAGAGTGG
si-EHBP1L1-2	TGCTATTTTACATCGATTCTACC
shEHBP1L1-1	ACCAGTATGAAACCAAAGAGTGG
shEHBP1L1-2	TGCTATTTTACATCGATTCTACC

Table S4. The primers used in this study.

**Name**	**Forward**	**Reverse**
**human genes**		
EHBP1L1	CTGTTACCACGAGCTAGTGTTG	TTCCGACGGGTCCATACCA
CD274	TGGCATTTGCTGAACGCATTT	TGCAGCCAGGTCTAATTGTTTT
GAPDH	TGCACCACCAACTGCTTAGC	GGCATGGACTGTGGTCATGAG
CXCL9	CCAGTAGTGAGAAAGGGTCGC	AGGGCTTGGGGCAAATTGTT
CXCL10	GTGGCATTCAAGGAGTACCTC	TGATGGCCTTCGATTCTGGATT
IRF1	ATGCCCATCACTCGGATGC	CCCTGCTTTGTATCGGCCTG
IRF9	GCCCTACAAGGTGTATCAGTTG	TGCTGTCGCTTTGATGGTACT
**murine genes**		
Ehbp1l1	GAAGGAACGGATCGCTCTGG	CCACGACCAGAGAAAGGGAC
Cd274	AGTATGGCAGCAACGTCACG	TCCTTTTCCCAGTACACCACTA
Gapdh	AGGTCGGTGTGAACGGATTTG	GGGGTCGTTGATGGCAACA
